# Efficacy of a WeChat-Based Multimodal Digital Transformation Management Model in New-Onset Mild to Moderate Hypertension: Randomized Clinical Trial

**DOI:** 10.2196/52464

**Published:** 2023-12-04

**Authors:** Yijun Wang, Fuding Guo, Jun Wang, Zeyan Li, Wuping Tan, Mengjie Xie, Xiaomeng Yang, Shoupeng Duan, Lingpeng Song, Siyi Cheng, Zhihao Liu, Hengyang Liu, Jiaming Qiao, Yueyi Wang, Liping Zhou, Xiaoya Zhou, Hong Jiang, Lilei Yu

**Affiliations:** 1 Department of Cardiology Renmin Hospital of Wuhan University Wuhan China; 2 Institute of Molecular Medicine Renmin Hospital of Wuhan University Wuhan China; 3 Hubei Key Laboratory of Autonomic Nervous System Modulation Wuhan China; 4 Taikang Center for Life and Medical Sciences Wuhan University Wuhan China; 5 Department of Cardiology The First Affiliated Hospital of Bengbu Medical College Bengbu China

**Keywords:** digital health care, mHealth, mobile health, apps, applications, controlled trials, digital transformation, precision, multimodal, precision medicine, hypertension, blood pressure, WeChat, social media, self-management, mobile phone

## Abstract

**Background:**

The advantages of multimodal digitally transformed mobile health management for patients diagnosed with mild to moderate hypertension are not yet established.

**Objective:**

We aim to evaluate the therapeutic benefits of a novel WeChat-based multimodal digital transforming management model in mobile health blood pressure (BP) management.

**Methods:**

This randomized controlled clinical trial included 175 individuals with new-onset mild to moderate hypertension who were admitted to our center between September and October 2022. The patients were randomly assigned to either the multimodal intervention group (n=88) or the usual care group (n=87). The primary composite outcome was home and office BP differences after 6 months. The major secondary outcomes were 6-month quality-of-life scores, including the self-rating anxiety scale, self-rating depression scale, and Pittsburgh Sleep Quality Index.

**Results:**

The mean home BP decreased from 151.74 (SD 8.02)/94.22 (SD 9.32) to 126.19 (SD 8.45)/82.28 (SD 9.26) mm Hg in the multimodal intervention group and from 150.78 (SD 7.87)/91.53 (SD 9.78) to 133.48 (SD 10.86)/84.45 (SD 9.19) mm Hg in the usual care group, with a mean difference in systolic blood pressure and diastolic blood pressure of –8.25 mm Hg (95% CI –11.71 to –4.78 mm Hg; *P*<.001) and –4.85 mm Hg (95% CI –8.41 to –1.30 mm Hg; *P*=.008), respectively. The mean office BP decreased from 153.64 (SD 8.39)/93.56 (SD 8.45) to 127.81 (SD 8.04)/ 82.16 (SD 8.06) mm Hg in the multimodal intervention group and from 151.48 (SD 7.14)/(91.31 (SD 9.61) to 134.92 (SD 10.11)/85.09 (SD 8.26) mm Hg in the usual care group, with a mean difference in systolic blood pressure and diastolic blood pressure of –9.27 mm Hg (95% CI –12.62 to –5.91 mm Hg; *P*<.001) and –5.18 mm Hg (95% CI –8.47 to –1.89 mm Hg; *P*=.002), respectively. From baseline to 6 months, home BP control <140/90 mm Hg was achieved in 64 (72.7%) patients in the multimodal intervention group and 46 (52.9%) patients in the usual care group (*P*=.007). Meanwhile, home BP control <130/80 mm Hg was achieved in 32 (36.4%) patients in the multimodal intervention group and 16 (18.4%) patients in the usual care group (*P*=.008). After 6 months, there were significant differences in the quality-of-life total and graded scores, including self-rating anxiety scale scores (*P*=.04), self-rating depression scale scores (*P*=.03), and Pittsburgh Sleep Quality Index scores (*P*<.001), in the multimodal intervention group compared with the usual care group.

**Conclusions:**

The WeChat-based multimodal intervention model improved the BP control rates and lowered the BP levels more than the usual care approach. The multimodal digital transforming management model for hypertension represents an emerging medical practice that utilizes the individual’s various risk factor profiles for primary care and personalized therapy decision-making in patients with hypertension.

**Trial Registration:**

Chinese Clinical Trial Registry ChiCTR2200063550; https://www.chictr.org.cn/showproj.html?proj=175816

## Introduction

Hypertension is one of the leading causes of disability and premature death worldwide, including in China [1-3]. Studies have shown that well-documented risk factors such as age, smoking, obesity, emotion, and dietary habits could accelerate the evolution of essential hypertension and prognosis [4-6]. Increased awareness, treatment, and medication adherence can help prevent and control hypertension among Chinese adults aged 35-75 years [2]. Given the prevalence of hypertension among the Chinese population, it’s crucial to educate the public on hypertension awareness and the importance of daily management.

With technical advancements in modern telecommunication, more digitalized risk factor management and mobile communication technology for hypertension management have been made available [7-16]. Previous studies have yielded telemonitoring and self-management as a significant novel addition to the control of hypertension in primary care [12-14]. Further, the digital therapeutics system, which includes facilitating the implementation and promoting the effectiveness of lifestyle modification information and behaviors, serves as a valuable addition to the existing initiatives for patients with hypertension, especially in the early stage [7-11]. Recently, a study indicated that public health benefits may be generated across diverse populations according to multiple modes of intervention delivery [17]. Combined with these interventions, the online platform can be beneficial for treating hypertension in the community.

The effectiveness of blood pressure (BP) interventions using various forms of mobile health (mHealth) management varies greatly among patients, which highlights the high interindividual variability. Hence, there is an urgent need to develop a mHealth care system that can leverage digital information to account for individual differences and assess the risk of hypertension based on multimodal data, enabling personalized and tailored health care management. Notably, the popularity of WeChat in China is increasing; its unique combination of user-friendly features and extensive social networking capabilities make it a valuable tool for promoting hypertension management and healthy lifestyles among the Chinese population [18,19]. However, studies comprehensively evaluating personalized hypertension management (which focuses on individual differences, customized approach, and incorporation of multimodal data, digital transformation, and multimodal intervention) using rigorous assessments to determine its effectiveness are scarce. Therefore, in this prospective, randomized, controlled clinical trial, we aim to evaluate the effectiveness of the novel WeChat-based multimodal intervention in the treatment of Chinese patients with new-onset hypertension, who are currently not on antihypertensive therapy.

## Methods

### Study Design and Participants

This prospective, randomized, controlled clinical trial was conducted at the Renmin Hospital of Wuhan University between September and October 2022 and followed up on April 2023.

The recruitment continued until the end of October 2022, and the planned sample size was achieved. The inclusion criteria were as follows: (1) age 20-80 years, (2) first diagnosis of essential hypertension (BP measured at home 3 times on different days; systolic blood pressure [SBP] >140 mm Hg or diastolic blood pressure [DBP] >90 mm Hg), (3) received no antihypertensive medications before enrollment, (4) used smartphones every day, and (5) considered appropriate to undergo lifestyle modification for 6 months. Patients with suspected secondary hypertension or hypertensive emergency were excluded. Multimedia Appendix 1 summarizes the inclusion and exclusion criteria.

### Randomization and Masking

Using an independent web-based block randomization system, participants were randomly assigned with equal probability to the multimodal intervention group or the usual care control group. Randomization was stratified by general practice with minimization for sex, age (>60 or ≤60 years), BMI (<30 or ≥30 kg/m^2^), baseline SBP (<160 mm Hg vs ≥160 mm Hg), and the presence or absence of diabetes and coronary artery disease. The patients in both trial groups received optimal therapy, as recommended by experienced clinicians, which contained primary lifestyle modifications and standardized management for hypertension.

### Ethical Considerations

This study’s protocol was approved by the institutional review board of the Renmin Hospital of Wuhan University (approval WDRY2022-K175) and registered at the Chinese Clinical Trial Registry (ChiCTR2200063550). The trial was also conducted in accordance with the Good Clinical Practice guidelines, the Declaration of Helsinki, and all applicable Chinese laws and guidelines. Written informed consent was obtained from each patient before inclusion. The trial protocol adhered to the CONSORT (Consolidated Standards of Reporting Trials) reporting guidelines (Multimedia Appendix 3).

### Procedures

The baseline data set, which contained sociodemographic features, behavioral patterns, and past medical histories, was obtained from the multimodal intervention and control groups during the recruitment process. A baseline standardized questionnaire survey, including the self-rating anxiety scale (SAS), self-rating depression scale (SDS), and Pittsburgh Sleep Quality Index (PSQI), was conducted by trained professionals during face-to-face interviews. Multimedia Appendix 1 contains the data acquisition details of this study’s protocol.

All patients were provided with information regarding lifestyle modification for hypertension management by physicians, which was in line with the Chinese hypertension management guidelines [20]. Broadly, in the multimodal intervention group, patients engaged in the digital transformation of personally controlled health information, which encompasses daily BP measurements, lifestyle modifications, daily activities, and progressions on the prescribed program. Moreover, using WeChat as a communication platform, patients were able to provide their physicians with weekly updates on their health condition encompassing BP, physical activity, dietary habits, and sleep patterns. WeChat served as a convenient platform for patients and clinicians to view online BP information through a visual interface. As a result, the patients and clinicians were engaged in a shared decision-making process to determine individual hypertension management. The main components of the intervention were personalized lifestyle modification programs via WeChat, complemented by the evaluation and systematization of the presence of various risk factors according to baseline data and online health information. Clinicians were able to offer customized online health education that was tailored to the individual patient’s specific circumstances, risk factors, interests, and objective conditions. By setting individual difference factors, patients were also able to schedule interactive support for intensive and personalized lifestyle modifications and modifiable risk factors through WeChat.

Online multimodal health education includes internet and online communication, text-based health education, medical graphic narratives, and animation-supported health education. The purpose of online health education is to make health education easier and more accessible to patients and bridge the existing knowledge gap between clinicians and patients. In the context of online health education, program health education, lifestyle modifications, and modifiable risk factors are fundamental elements, which include how to choose an appropriate diet, expert guidance on exercise patterns and timing, and knowledge regarding the hazards of smoking and drinking, improved sleep patterns, and psychological care. Multimedia Appendix 1 details the description of the intervention activities.

The multimodal intervention group comprises cardiologists who possess extensive expertise in their respective professional domains. They underwent standardized training to ensure the consistent provision of high-quality health education. We employed standardized health education materials to minimize variations among doctors. Further, regular quality control and feedback mechanisms are implemented to ensure the efficacy of the intervention. Involving patients in the digital transformation of health information is a crucial step as it empowers them to better comprehend and manage their health conditions. To guarantee the effectiveness of our intervention, we adopted several strategies: providing detailed guidance and support for patients to correctly use WeChat-based multimodal intervention, regularly monitoring patients’ usage while offering feedback and suggestions, as well as conducting biweekly evaluations on effectiveness to monitor and enhance outcomes.

Regarding the follow-up schedule, patients in the multimodal intervention group were followed up online once a week and as outpatients at 3 and 6 months. However, patients in the usual care group presented at baseline and were followed up as outpatients at 3 and 6 months. All patients were scheduled for morning home BP measurements every week and had to provide feedback to the clinical physician during follow-up. Accordingly, the clinical physician modifies the management based on the BP situation. These patients also had to refill the standardized questionnaire at the end of the trial. The principles of low-dose initiation, long-acting medication, combination therapy, and individualization were used according to the Chinese hypertension management guidelines [20], which consisted of routine care for the prevention and treatment of hypertension by physicians.

### Measurements

Eligible patients were taught how to employ automated electronic sphygmomanometers, which were validated. All participants were scheduled to measure their home BP in their right arm every morning and every weekend according to standard recommendations [20,21]. A detailed description of the intervention activities is provided in Multimedia Appendix 1.

BMI (calculated as weight in kilograms divided by height in meters squared) was obtained from self-reported height and weight. Nonobese BMI was defined as <30 kg/m^2^, and obesity was defined as a BMI of ≥30 kg/m^2^ [22]. Psychometric psychological-related measures, including SAS [23], SDS [24], and PSQI [25], were administrated. SAS was used to assess the individual’s level of anxiety [23]. SDS was used to assess the severity of depression [24]. The PSQI, a self-reported questionnaire, was used to assess sleep quality and disturbances over a 1-month time interval [25]. Quality-of-life (QoL) scores and details regarding quality control are provided in Multimedia Appendix 1.

### Outcomes

Follow-up clinical outcome measurements were performed at 3 and 6 months. The primary composite outcome was home and office BP differences after 6 months. Major secondary outcomes were differences in QoL scores (SAS, SDS, and PSQI) after 6 months. Definitions of clinical outcomes and details regarding quality control are provided in Multimedia Appendix 1.

### Statistical Analysis

The sample size estimation was according to the test of 2 independent proportions, with a 2.5% type 1 error rate, an intracluster correlation coefficient of 0.01, and an 80% retention rate. We aimed to have 90% power to detect a 20% absolute difference in BP control rates between the intervention and usual care group at the 6-month follow-up. According to our calculation, at least 80 evaluable patients would be required per group for this study to achieve this power.

Normally distributed continuous variables were expressed as means and SDs, and medians and IQRs were otherwise presented. Categorical variables were analyzed using the chi-square test or Fisher exact test, while continuous variables were analyzed using a 2-tailed *t* test or Mann-Whitney test (as appropriate). The B*P* values at different time points were analyzed for intervention using repeated measures’ analysis of variance. The B*P* values at various time points during the intervention were analyzed using repeated measures’ analysis of variance. To assess the intervention effect, the disparity between the 2 groups was calculated by subtracting the difference in values at 6 months and the baseline for the control group from that of the intervention group. Subsequently, an independent *t* test was conducted to determine differences between pre-and postintervention data within each group. A *P* value below .05 indicates a statistically significant divergence in interventions observed among both groups. Further, we conducted exploratory analyses in predefined subgroups, including sex, age (>60 or ≤60 years), BMI (<30 or ≥30 kg/m^2^), currently smoking, currently drinking, baseline SBP (<160 mm Hg vs ≥160 mm Hg), and the presence or absence of diabetes and coronary heart disease. The data were analyzed using SPSS (version 23; IBM Corp) and R (version 4.2.2; R Foundation for Statistical Computing). A 2-sided *P*<.05 was considered statistically significant.

## Results

**Figure 1:**
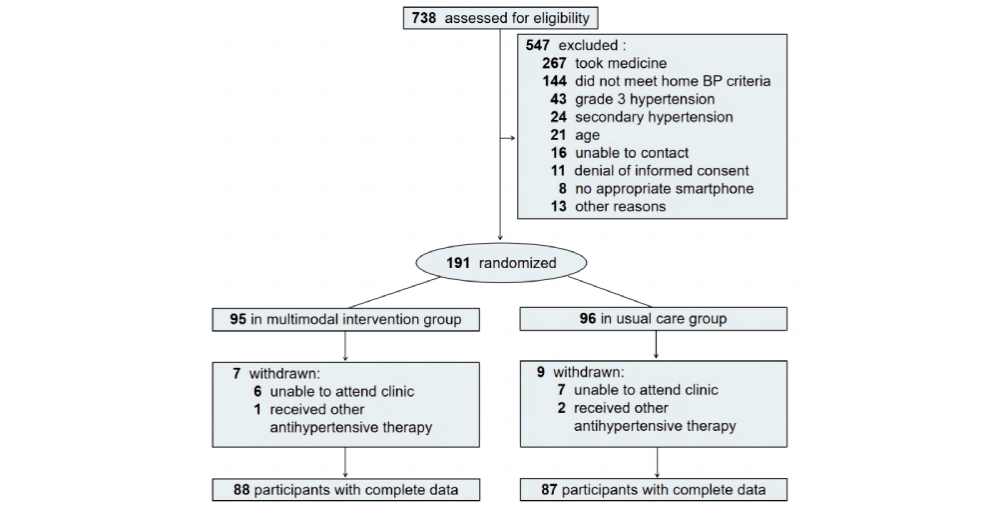
Screening, randomization, and follow-up flowchart. BP: blood pressure.

Participant recruitment and retention are presented in Figure 1. Of the 175 patients (mean age, 50.8, SD 14.2 years) with complete data, 81 (46.3%) were female and 94 (53.7%) were male. Participants randomized to multimodal intervention (n=88) had similar features to those randomized to usual care (n=87) at baseline. Table 1 indicates the baseline characteristics for multimodal intervention and usual care groups. Table 2 demonstrates a statistically significant difference between both groups in home SBP at 1, 2, 3, 4, 5, and 6 months (*P*<.001). Concurrently, home DBP did not differ between both groups at 1, 2, 3, 4, 5, and 6 months (all *P*>.05). From baseline to the first 6 months of intervention, there is a marked decline in home SBP level in the multimodal intervention group from 151.74 mm Hg to 126.19 mm Hg (*P*<.001) and from 150.78 mm Hg to 133.48 mm Hg in the usual care group (*P*<.001; Table 2). Similarly, from baseline to the first 6 months, home DBP level decreased from 94.22 mm Hg to 82.28 mm Hg and from 91.53 mm Hg to 84.45 mm Hg in the multimodal intervention and usual care groups, respectively (*P*<.001; Table 2).

**Table 1:** Demographic and baseline clinical characteristics at baseline.

Characteristics		Intervention (n=88)	Usual care (n=87)
Male, n (%)		47 (53.4)	47 (54)
Age (years), mean (SD)		50.90 (14.21)	50.70 (14.31)
**Educational qualifications, n (%)**			
	Elementary school and below	22 (25)	18 (20.7)
	Middle school or high school	34 (38.6)	28 (32.2)
	Bachelor’s degree and above	32 (36.4)	41 (47.1)
Diabetes mellitus, n (%)		22 (25)	15 (17.2)
Coronary artery disease, n (%)		7 (8)	13 (14.9)
Cerebrovascular disease, n (%)		14 (15.9)	13 (14.9)
Hyperlipemia, n (%)		49 (55.7)	43 (49.4)
Currently smoking, n (%)		26 (29.5)	22 (25.3)
Currently drinking, n (%)		28 (31.8)	27 (31)
Family history of hypertension, n (%)		69 (78.4)	66 (75.9)
**Good sleep quality, n (%)**			
	Very often	5 (5.7)	3 (3.4)
	Often	36 (40.9)	36 (41.4)
	Sometimes	44 (50)	43 (49.4)
	Rarely	3 (3.4)	5 (5.7)
Sleep duration (hours/day), mean (SD)		6.51 (1.17)	6.45 (1.52)
Sedentary time (hours/day), mean (SD)		5.63 (2.20)	5.98 (2.34)
SDS^a^ scores, mean (SD)		47.42 (7.97)	45.69 (7.79)
**SDS group, n (%)**			
	Good	64 (72.7)	65 (74.7)
	Mild depression	24 (27.3)	22 (25.3)
SAS^b^ scores, mean (SD)		51.51 (9.53)	49.35 (11.05)
**SAS group, n (%)**			
	Normal	39 (44.3)	44 (50.6)
	Mild	28 (31.8)	22 (25.3)
	Moderate	21 (23.9)	21 (24.1)
PSQI^c^ scores, mean (SD)		8.76 (2.63)	9.32 (3.41)
**PSQI group, n (%)**			
	Normal	6 (6.8)	10 (11.5)
	Mild	58 (65.9)	49 (56.3)
	Moderate	23 (26.1)	26 (29.9)
	Severe	1 (1.1)	2 (2.3)
Baseline SBP^d^ (mm Hg), mean (SD)		151.74 (8.02)	150.78 (7.87)
Baseline DBP^e^ (mm Hg), mean (SD)		94.22 (9.32)	91.53 (9.78)
Baseline BMI (kg/m^2^), mean (SD)		24.99 (3.66)	24.75 (3.50)

^a^SDS: self-rating depression scale.

^b^SAS: self-rating anxiety scale.

^c^PSQI: Pittsburgh Sleep Quality Index.

^d^SBP: systolic blood pressure.

^e^DBP: diastolic blood pressure.

**Table 2:** Mean BP^a^ and QoL^b^ scores.

Characteristics			Intervention (n=88), mean (SD)		Usual care (n=87), mean (SD)		*t* test		*P* value
**SBP^c^, mean (SD)**									
	Baseline	151.74 (8.02)		150.78 (7.87)		0.797 (173)		.43	
	One month	135.68 (7.53)		136.68 (8.4)		0.827 (173)		.41	
	Two months	134.75 (7.55)		140.25 (9.48)		4.245 (173)		<.001	
	Three months	128.81 (7.71)		134.52 (10.00)		4.227 (173)		<.001	
	Four months	129.68 (8.62)		136.86 (10.53)		4.939 (173)		<.001	
	Five months	125.28 (8.17)		132.55 (10.52)		5.107 (173)		<.001	
	Six months	126.19 (8.45)		133.48 (10.86)		4.957 (173)		<.001	
*F* test (*df*)			231.876 (87)		69.386 (86)				
*P* value			<.001		<.001				
**DBP^d^, mean (SD)**									
	Baseline	94.22 (9.32)		91.53 (9.78)		1.861 (173)		.06	
	One month	83.42 (8.31)		85.20 (9.73)		1.298 (173)		.20	
	Two months	86.13 (8.90)		86.67 (9.71)		0.385 (173)		.70	
	Three months	86.16 (9.07)		87.35 (9.79)		0.831 (173)		.41	
	Four months	85.60 (9.39)		84.52 (9.81)		0.748 (173)		.46	
	Five months	83.55 (9.25)		86.07 (9.13)		1.816 (173)		.07	
	Six months	82.28 (9.26)		84.45 (9.19)		1.551 (173)		.12	
*F* test (*df*)			38.391 (87)		10.101 (86)				
*P* value			<.001		<.001				
**BP over 6 months**, mean (SD)									
	SBP	133.16 (6.12)		137.88 (7.26)		4.647 (173)		<.001	
	DBP	85.91 (7.18)		86.54 (6.91)		0.592 (173)		.55	
**QoL total scores, mean (SD)**									
	Six-month SDS^e^ scores	40.92 (7.88)		43.56 (8.31)		2.159 (173)		.03	
	Six-month SAS^f^ scores	42.66 (8.92)		45.76 (10.40)		2.116 (173)		.04	
	Six-month PSQI^g^ scores	7.32 (2.14)		8.89 (2.85)		4.114 (173)		<.001	

^a^BP: blood pressure.

^b^QoL: quality-of-life.

^c^SBP: systolic blood pressure.

^d^DBP: diastolic blood pressure.

^e^SDS: self-rating depression scale.

^f^SAS: self-rating anxiety scale.

^g^PSQI: Pittsburgh Sleep Quality Index.

**Figure 2:**
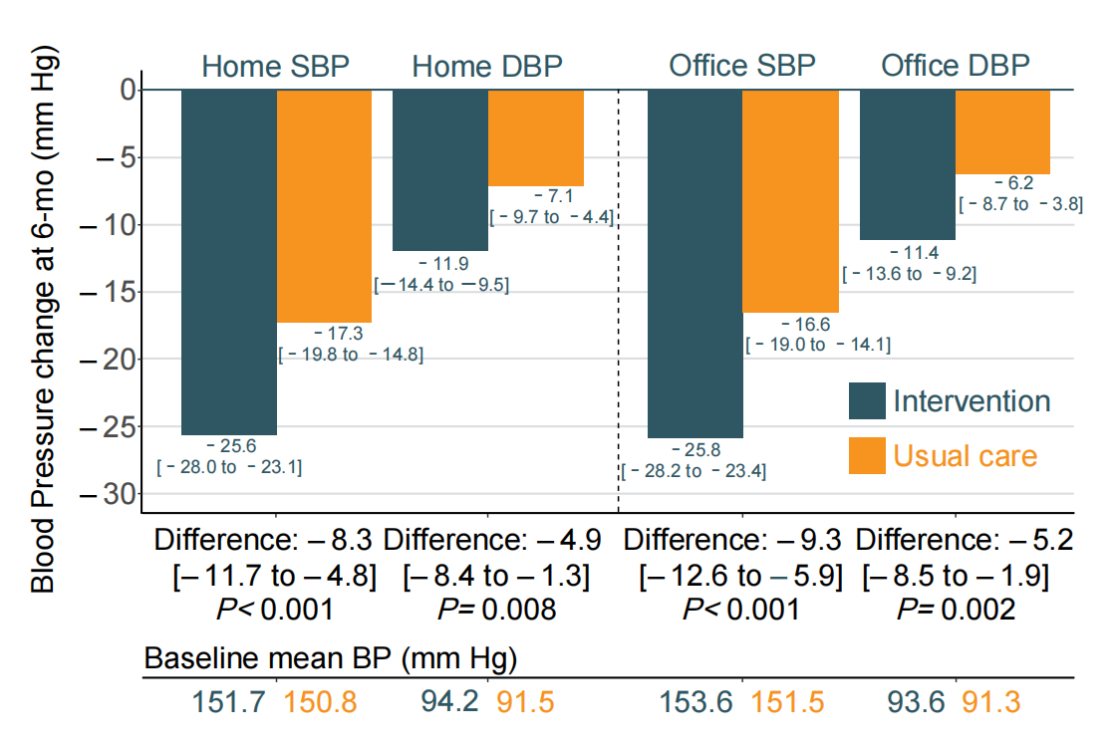
Changes from baseline to 6 months in BP. BP: blood pressure, DBP: diastolic blood pressure; SBP: systolic blood pressure.

The mean difference in home SBP change between the multimodal intervention and usual care groups was –6.67 mm Hg (95% CI –9.76 to –3.57 mm Hg) at 3 months (*P*<.001) and –8.25 mm Hg (95% CI –11.71 to –4.78 mm Hg) at 6 months (*P*<.001; Figure 2 and Table S1 in Multimedia Appendix 2). The mean difference in home DBP change between both groups was –3.87 mm Hg (95% CI –7.34 to –0.40 mm Hg) at 3 months (*P*=.03) and –4.85 mm Hg (95% CI –8.41 to –1.30 mm Hg) at 6 months (*P*=.008; Figure 2 and Table S1 in Multimedia Appendix 2). Likewise, both office SBP and DBP levels during the trial follow-up were lower in the multimodal intervention group than in the usual care group (Figure 2 and Table S2 in Multimedia Appendix 2).

Changes based on home BP and office SBP. Values are reported as the mean (95% CI) of the difference in change in BP between the multimodal intervention and usual care group.

From baseline to the first 6 months of intervention, the control rates of home BP (<140/90 mm Hg) reached 64 (72.7%) patients in the multimodal intervention group and 46 (52.9%) patients in the usual care group, and the intervention effect was significant (OR 2.377, 95% CI 1.265 to 4.464; *P*=.007; Figure S1 and Table S3 in Multimedia Appendix 2). Except for sex, diabetes mellitus, coronary heart disease, and baseline home SBP (<160 mm Hg or ≥160 mm Hg), there was a significant interaction relationship (Table S4 in Multimedia Appendix 2). Likewise, the control rates of home BP (<130/80 mm Hg) reached 32 (36.4%) patients in the multimodal intervention group and 16 (18.4%) patients in the control group, with a significant intervention effect (OR 2.536, 95% CI 1.266 to 5.080; *P*=.008; Figure S1 and Table S3 in Multimedia Appendix 2). The intervention’s effect on home BP control rate indicated a significant interaction with the sex subgroup, whereas no interaction relationship existed for the other subgroups. Similarly, better office BP control rates (<140/90 mm Hg and <130/80 mm Hg) were observed in the multimodal intervention than in the usual care group (Figure S2 in Multimedia Appendix 2).

**Figure 3:**
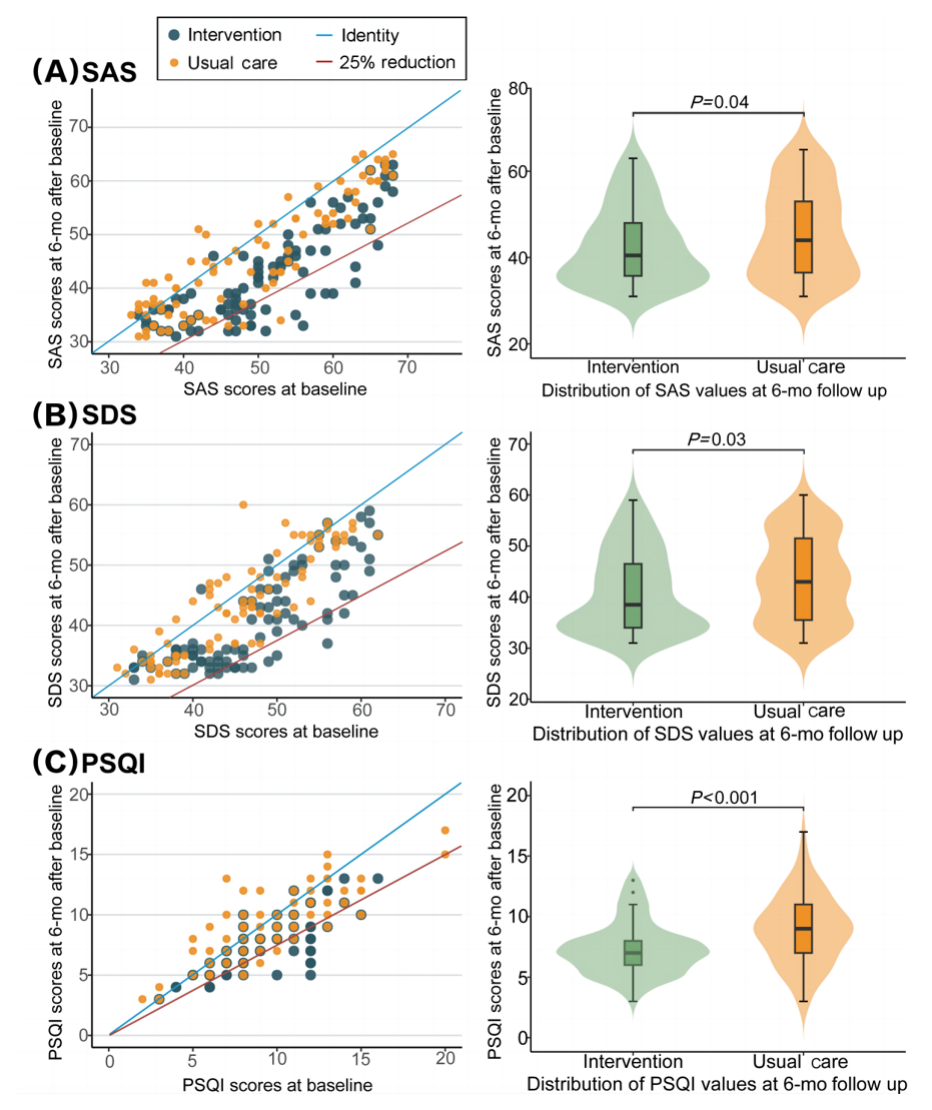
SAS scores, SDS scores, and PSQI scores at baseline versus 6 months and violin plots of SAS scores, SDS scores, and PSQI scores at 6 months in the multimodal intervention and usual care groups. SAS: self-rating anxiety scale; SDS: self-rating depression scale; PSQI: Pittsburgh Sleep Quality Index.

After 6 months, there were statistically significant differences between the multimodal intervention group and the usual care group, wherein improved QoL total and grading scores, including SDS, SAS, and PSQI scores, were observed in the multimodal intervention group (Figure 3 and Table 2).

In both scatterplots (left panels), the diagonal blue line divides participants who improved in SAS scores, SDS scores, and PSQI scores at 6 months (below the line) from those who worsened (above the line). The diagonal red line divides participants who achieved at least a 25% reduction from baseline SAS scores, SDS scores, and PSQI scores at 6-months follow-up (below the line) from those who did not achieve it (above the line), to illustrate the primary outcome. In the violin plots, the horizontal line in the box indicates the median, the upper and lower ends of the boxes indicate the 25th and 75th quartiles and the whiskers indicate the highest and lowest values within 1.5 times the IQR of SAS scores, SDS scores, and PSQI scores. The wider shaded areas in the violin plots indicate a higher probability that members of the population will take on the given value; thinner areas indicate a lower probability. The SAS median score was 50 (IQR 42.25-59) for the multimodal intervention group and 49 (IQR 39-59) for the usual care group. The SDS median score was 38.5 (IQR 34-46) for the multimodal intervention group and 43 (IQR 37-50) for the usual care group. The PSQI median score was 8 (IQR 7-11) for the multimodal intervention group and 9 (IQR 7-11) for the usual care group.

Furthermore, the SAS score at 6 months in the multimodal intervention group was –5.34 (95% CI –6.98 to –3.71; *P*=.04; Figure 3A and Table S6 in Multimedia Appendix 2). The mean difference in SDS score change between the 2 groups was –4.41 (95% CI –5.76 to –3.07) at 6 months (*P*=.03; Figure 3B and Table S6 in Multimedia Appendix 2). Meanwhile, the mean difference for PSQI score of –1.00 points (95% CI –1.53 to –0.47) exhibited an estimated difference in means between the multimodal intervention and usual care groups at 6 months (*P*<.001; Figure 3C and Table S6 in Multimedia Appendix 2).

No serious cardiovascular disease events were correlated with either of the groups (Table S9 in Multimedia Appendix 2). Three patients in the multimodal intervention group experienced antihypertensive drug-related adverse effects: 1 (1.14%) suffered from stomach discomfort and 2 (2.27%) developed dizziness. In the usual care group, four patients suffered from antihypertensive drug-related adverse effects: 1 (1.15%) suffered from stomach discomfort and 3 (3.45%) developed dizziness (Table S9 in Multimedia Appendix 2).

## Discussion

### Principal Findings

This randomized clinical trial demonstrated that the novel WeChat-based mHealth management for BP using digital transformation of baseline data and online health information was effective in the multimodal intervention group as compared to the usual care group. This system was conducive to better BP control rates (<140/90 mm Hg and <130/80 mm Hg), and lower SBP levels. Concurrently, it enhanced lifestyle modifications and contributed to constant improvement in the QoL of Chinese patients with new-onset hypertension.

### Comparison With Prior Work

Indisputably, lifestyle modifications and modifiable risk factors in clinical practice facilitate hypertension management; however, clinically, hypertension patients usually cannot achieve the desired goal of BP control [1-3]. In general, this phenomenon is not accidental and is usually attributed to the lack of awareness regarding hypertension, inadequate treatment, or lack of medication adherence, which contributes to poor prevention and control of hypertension [2]. With increasing technological advancements, the multidisciplinary care system is constantly changing and improving. The MyHEART (My Hypertension Education And Reaching Target) randomized clinical trial, which incorporated components of telephone coaching and home BP monitoring with standard clinical care, found that this approach could assist young adults with uncontrolled hypertension in lowering their BP through lifestyle modifications [26]. A cluster randomized clinical trial demonstrated the benefit of integrating diverse intervention components using smartphones in patients with hypertension and may be conducive across diverse populations [17]. Alternatively, data from the HERB-DH1 (HERB Digital Hypertension 1) pivotal trial provided new insights into the management of essential hypertension based on comprehensive digital therapeutic interventions [9]. The above potential effects enable a variety of patient subgroups to better integrate into primary care–based chronic disease management programs and stimulate the harmonious development of individuals and BP management. These variations in findings could be attributed to the fact that previous studies were conducted using different designs, interventions, and time frames. Therefore, literature supporting comprehensive and systematic evidence-based risk assessment aids in the digital transformation of multimodal intervention and personalized therapy are scarce.

As a leading social media platform in China, WeChat can serve as a powerful tool for managing hypertension and promoting a healthy lifestyle among the Chinese population [27]. Due to its user-friendly interface and extensive network of users, WeChat offers a unique advantage in delivering health-related information and support to individuals with hypertension [27]. In this study, we developed a WeChat-based multimodal digital transforming management (MDTM) model that featured the following: visual and personalized model-based risk assessment; patient-centered, dynamic, real-time, personalized home telemonitoring; and digital transformation of multimodal intervention for online health education. In the multimodal intervention group, our findings were consistent with those reported in previous studies, which found a trend toward a significant decrease in both morning home SBP and achieved better BP control from baseline to the first 6 months of intervention [9,17].

In the MDTM model, daily BP changes and personally controlled health information, which consisted of lifestyle modifications and daily activities, were visualized for the clinicians and patients. This enabled a complete assessment of the potential risk factors and provided personalized health education and timely shared decision-making. Overall, the results for BP control in our study were similar to those reported in previous studies [28]. Specifically, we added digital transformation of multimodal intervention, which enabled personalized intervention, and combined with multiple treatment modalities, including visual BP health analytics, BP self-monitoring reminders, medication dosing reminders, digital medical knowledge cards, cardiovascular disease educational animation, health lessons, and online video communication between physicians and patients. The MDTM model heightened clinician awareness to establish a favorable vibe for patients with hypertension. Further, it enabled patients to fully understand hypertension, increased their awareness, and encouraged lifestyle behavior implementation, which helped accelerate self-management in hypertension. When conducting health education using a multimodal intervention model, the system adopts different intervention methods according to personal information, such as age, sex, interests, and living environment. This allows patients to learn health knowledge in a pleasant atmosphere and develop healthy behaviors. For instance, in older patients, health education can be delivered using animations and videos to attract their attention and enhance their memory. This can be further complemented by providing them with interaction videos with the clinician. These approaches have the potential to better meet the needs and interests of diverse groups of patients and enable them to actively learn health knowledge, form a healthy lifestyle, and promote personal and social health development. Of all the benefits, the most appealing aspect of the MDTM model was its clinical applicability and user-friendliness, making it accessible to a diverse range of individuals. This ease of use facilitates the provision of health education and helps bridge the knowledge gap between health care providers and different populations. Further, the MDTM model is cost-effective; it can be supplied as either “software as a service” or cloud services. Studies evaluating QoL based on standardized questionnaires to provide efficacy evaluation of mHealth management are limited. In line with previous studies, the results of our trial not only confirmed that the MDTM model can improve symptoms of depression and anxiety in patients with hypertension [29] but also shows that it can improve sleep quality. This MDTM model not only enhances cost-effectiveness and reduces clinician-patient contact time, but also provides patients with ample access to knowledge and resources for lifestyle and behavior modification. This results in the provision of more pertinent and convenient mental health information, thereby improving the quality of care. Furthermore, the MDTM model can help patients overcome psychological problems, which enhances their mental health and sleep quality and promotes the harmonious development of individuals with hypertension. At an individual level, the MDTM model serves as a platform that provides psychoeducation and medication management, lifestyle and behavioral modification, rechanneling emotions, improvement of sleep quality, and symptom tracking, thereby amplifying the benefits of existing therapy. Moreover, the MDTM model is convenient and accessible, particularly for patients seeking mental health support, as it offers the added benefit of accessing mental and sleep-quality health care from the comfort of one’s own home. Further, the MDTM model offers increased privacy and confidentiality compared to in-person visits, making individuals feel more comfortable and open during their sessions. Nonetheless, to ensure the robustness of our results, the need for replication and further validation of our findings in larger well-defined populations, as well as for cause-specific outcomes, is warranted.

### Limitations

This study has some limitations that should be considered when interpreting the results. First, the effectiveness of the MDTM model needs to be validated in a larger or well-defined population. Second, given the relatively short follow-up duration, we were not certain whether the improvement in lifestyle modification, QoL, and BP control rate would be sustained. Third, the MDTM model consists of multiple component interventions; however, the nature of multimodal interventions has not been explored further. Future research regarding the effectiveness of these intervention measures and the mediating and moderating factors of their implementation is warranted to determine the impact of any specific component and further improve their effectiveness, coverage, uptake, and feasibility. Fourth, self-reported data on standardized questionnaires made recall bias inevitable when assessing concomitant subjectivity regarding the method and weakness of the measures. Fifth, during the long-term upgrading and optimization process of digital health management, further education of health care professionals, researchers, and the public is needed to help patients understand the working principles of digital health management and bridge the existing knowledge gap.

### Conclusions

Our study presented a novel hypertension management model that could provide integrated, fine-grained management of diseases online and offline, thus promoting doctor-patient communication through multidimensional intervention. The model can enhance a comprehensive understanding of hypertension and improve medication adherence among patients. This model is an advanced step toward achieving patient-centered preventive and therapeutic care and chronic disease management, ultimately contributing to improving people’s health.

## Appendix

Multimedia Appendix 1 Protocol summary.

Multimedia Appendix 2 More data analysis.

Multimedia Appendix 3 CONSORT-eHEALTH Checklist (V1.6.1).

## Abbreviations

BP: blood pressure
CONSORT: Consolidated Standards of Reporting Trials
DBP: diastolic blood pressure
HERB-DH1: HERB Digital Hypertension 1
MDTM: multimodal digital transforming management
mHealth: mobile health
MyHEART: My Hypertension Education And Reaching Target
PSQI: Pittsburgh Sleep Quality Index
QoL: quality-of-life
SAS: self-rating anxiety scale
SBP: systolic blood pressure
SDS: self-rating depression scale

## Data Availability

Individual participant data that underlie the results reported in this paper, after deidentification can be obtained from the corresponding author upon reasonable request.
