# Supplementary material for: Efficacy of a WeChat-Based Multimodal Digital Transformation Management Model in New-Onset Mild to Moderate Hypertension: Randomized Clinical Trial
Source: J Med Internet Res. 2023 Dec 4;25:e52464. doi: 10.2196/52464 (PMC10728790; doi:10.2196/52464)
Supplement: Multimedia Appendix 1 [file jmir_v25i1e52464_app1.docx]

**The Efficacy of a WeChat-Based Multimodal Digital Transformation Management Model in New-Onset Mild-to-Moderate Hypertension:**

**Protocol for a Randomized Controlled Trial**

**Title:** The Efficacy of a WeChat-Based Multimodal Digital Transformation Management Model in New-Onset Mild-to-Moderate Hypertension: A Randomized Clinical Trial

**Brief Title:** Benefits of multimodal management of hypertension

***Correspondence:**

**Lilei Yu, MD, PhD, FESC, FACC**

Department of Cardiology, Renmin Hospital of Wuhan University,

No.238 Jiefang Road, Wuhan City, Hubei Province 430060, P.R. China,

Tel: +86 27 88041911; Fax: +86 27 88040334,

E-mail: [lileiyu@whu.edu.cn](mailto:lileiyu@whu.edu.cn)

**List of Abbreviations**

| **AE** | Adverse event |
| --- | --- |
| **APP** | Application |
| **BMI** | Body mass index |
| **BP** | Blood pressure |
| **CI** | Confidence intervals |
| **Cm** | Centimeters (used in measurements of height) |
| **CVD** | Cardiovascular disease |
| **DBP** | Diastolic blood pressure |
| **EHR** | Electronic health record |
| **IQRs** | Interquartile ranges |
| **IRB** | Institutional Review Board |
| **Kg** | Kilograms (used in measurements of weight) |
| **Kg/m^2^** | Kilograms per meters squared (used in measurements of body mass index) |
| **MDTM** | Multimodal Digital Transformation Management |
| **PSQI** | Pittsburgh Sleep Quality Index |
| **SAS** | Self-rating anxiety scale |
| **SAE** | Serious adverse event |
| **SBP** | Systolic blood pressure |
| **SD** | Standard deviation |
| **SDS** | Self-rating depression scale |

**Protocol Summary**

| **Short Title** | Multimodal digital transforming management and hypertension. |
| --- | --- |
| **Objectives** | The establishment of real-time, dynamic, accurate, and personalized hypertension health management is facilitated through the integration of multimodal data utilizing electronic health systems. |
| **Study Design** | This study is a prospective, single-center, interventional, randomized controlled study . |
| **Ethical Registration Number** | WDRY2022-K175 |
| **Clinical Research Registration Number** | ChiCTR2200063550 |
| **Study Center** | Renmin hospital of Wuhan University, China Wuhan |
| **Primary Outcome** | The primary outcome was the difference in home and office BP after 6-months. |
| **Secondary Outcome** | 6 months quality-of-life scores, contained self-rating anxiety scale (SAS), the self-rating depression scale (SDS), and the Pittsburgh Sleep Quality Index (PSQI). |
| **Population** | 80 patients with new-onset hypertension will be recruited and randomized to the multimodal intervention group (n=80) or the usual care group (n=80). |
| **Study Duration** | 6 months |
| **Follow-up visit** | 3 months, 6 months |
| **Intervention Group** | Multimodal data, digital transformation, and multimodal intervention.  Patients receive the MDTM model and standardized management from experienced clinicians including outpatient medication and lifestyle modifications. |
| **Control Group** | Usual care.  Patients receive standardized management from experienced clinicians including outpatient medication and lifestyle modifications. |
| **Main Inclusion Criteria** | The first diagnosis of essential hypertension ( BP measured at home three times on different days with a systolic BP > 140 mmHg or diastolic BP > 90 mmHg ) , had not taken antihypertensive medications before enrollment. |
| **Main Exclusion Criteria** | Exclusion of patients with suspected secondary hypertension or hypertension emergency. |
| **Randomization** | After fulfilling all eligibility criteria, subjects will be randomized 1:1 to receive multimodal intervention or usual care. |
| **Statistical Methodology** | All patients who completed the 6 months follow-up were included in the analysis for the primary outcome and secondary outcome. Normally distributed continuous variables are expressed as mean ± standard deviation (SD), and medians and interquartile ranges (IQRs) are otherwise presented. Categorical variables were analyzed using the χ2 test or the Fisher exact test, while continuous variables were conducted to analyze with the t-test or Mann-Whitney test (as appropriate). The blood pressure values at various time points during the intervention were analyzed using repeated measures analysis of variance. To assess the intervention effect, the disparity between two groups was calculated by subtracting the difference in values at 6 months and baseline for the control group from that of the intervention group. Subsequently, an independent t-test was conducted to determine differences between pre- and post-intervention data within each group. A p-value below 0.05 indicates a statistically significant divergence in interventions observed among both groups. Additionally, we conducted exploratory analyses in predefined subgroups, including sex, age (>60 or ≤60 years), BMI (＜30 or ≥30 kg/m2), current smoking, current drinking, baseline systolic BP (＜160 mm Hg vs ≥160 mm Hg) and presence or absence of diabetes as well as coronary heart disease. The study was not powered to show differences in the incidence of clinical events such as death, acute myocardial infraction, percutaneous coronary intervention , or drug type for monotherapy between multimodal intervention group and the usual care group, for which reason no statistical testing was performed regarding these variables. |
| **Safety** | Adverse events will be summarized for each group.  Adverse effects due to medication or lifestyle changes during/post intervention will be documented. |
| **Result** | Of the 175 patients (mean age, 50.8 years) with complete data, 81 were female (46.3%) and 94 were male (53.7%). Participants randomized to multimodal intervention (n = 88) had similar features to those randomized to usual care (n = 87) at baseline. The mean home BP decreased from 151.74/94.22 to 126.19/82.28 mmHg in the multimodal intervention group and from 150.78/91.53 to 133.48/84.45 mmHg in the usual care group, with a mean difference in systolic BP and diastolic BP of -8.25 mmHg (95% confidence interval [CI] -11.71 to -4.78 mmHg) and -4.85 mmHg (95% CI -8.41 to -1.30 mmHg), respectively. The mean office BP decreased from 153.64/93.56 to 127.81/82.16 mmHg in the multimodal intervention group and from 151.48/91.31 to 134.92/85.09 mmHg in the usual care group, with a mean difference in systolic BP and diastolic BP of -9.27 mmHg (95% confidence interval [CI] -12.62 to -5.91 mmHg) and -5.18 mmHg (95% CI -8.47 to -1.89 mmHg), respectively. From baseline to 6 months, home BP control <140/90 mmHg was achieved in 64 patients (72.7%) in the multimodal intervention group and 46 patients (52.9%) in the usual care group (*P* = 0.007). Meanwhile, home BP control <130/80 mmHg was achieved in 32 patients (36.4%) in the multimodal intervention group and 16 patients (18.4%) in the usual care group (*P* = 0.008). The 6-month QoL scores (SAS, SDS, and PSQI) suggested that patients in the multimodal intervention group benefitted from the program. |

**Contents**

[List of Abbreviations 1](#_Toc26977)

[Protocol Summary 2](#_Toc13095)

[1. Introduction and Rationale 1](#_Toc9713)

[1.1 Introduction 1](#_Toc3698)

[1.2 Rationale 2](#_Toc16063)

[2. Study Design and Purpose 4](#_Toc11034)

[2.1 Study Design 4](#_Toc32305)

[2.2 Study Purpose 4](#_Toc19625)

[2.2.1 Primary Objective 4](#_Toc6523)

[2.2.2 Secondary Objectives 5](#_Toc7178)

[3. Study Population 5](#_Toc25658)

[3.1 Enrollment Population 5](#_Toc15168)

[3.2 Inclusion and exclusion criteria. 5](#_Toc2524)

[3.3 Informed Consent 6](#_Toc15278)

[3.4 Early Withdrawal of subjects 7](#_Toc13255)

[3.5 Replacement of participant 8](#_Toc29194)

[3.6 Early termination of the study 8](#_Toc22304)

[3.7 Interim analyses 8](#_Toc6447)

[3.8 Screening, Randomization, and Follow-up Flowchart 8](#_Toc19517)

[4. Study Procedures 9](#_Toc8144)

[4.1 Randomization 9](#_Toc5518)

[4.2 Blinding 10](#_Toc32482)

[4.3 Intervention and Control 10](#_Toc20334)

[4.3.1 Intervention Strategy 10](#_Toc13217)

[4.3.2 Control group 13](#_Toc21298)

[4.4 Study Duration 13](#_Toc26356)

[4.4.1 Study Outcome 13](#_Toc3968)

[4.4.2 Clinical Assessments 14](#_Toc30937)

[4.4.3 Statistical Method 17](#_Toc10372)

[4.4.4 Sample Size Consideration 18](#_Toc22605)

[5.Data 18](#_Toc18120)

[5.1 Data Collection 18](#_Toc4934)

[5.2 Data Transmission 18](#_Toc29102)

[5.3 Data Processing 18](#_Toc10175)

[5.4 Data Storage 18](#_Toc31458)

[5.5 Data Sharing 19](#_Toc9703)

[5.6 Data Retention Period 19](#_Toc27200)

[5.7 Cancellation of Participation 19](#_Toc29929)

[5.8 Researcher Confidentiality 19](#_Toc5632)

[6. Safety 19](#_Toc22259)

[6.1 Adverse Events 19](#_Toc12581)

[6.1.1 Definition 19](#_Toc28252)

[6.1.2 Monitoring 20](#_Toc10882)

[6.1.3 Solution 20](#_Toc21789)

[6.2 Safety of Data Collection 21](#_Toc18646)

[6.3 Safety of WeChat Data 22](#_Toc8445)

[6.4 Study Discontinuation Rules 23](#_Toc5319)

[6.5 Safety Oversight Committee 23](#_Toc16919)

[7. Study Monitoring 23](#_Toc26573)

[7.1 Clinical Monitoring 23](#_Toc22187)

[7.1.1 Medication Reminders and Instructions 24](#_Toc28483)

[7.1.2 Lifestyle Intervention 24](#_Toc9813)

[7.2 Study Process Supervision 24](#_Toc11581)

[7.3 Adverse Event Monitoring 24](#_Toc18465)

[8. Ethical Considerations 24](#_Toc3282)

[9. Appendix 26](#_Toc21011)

[Appendix 1. Informed Consent 26](#_Toc3584)

[Appendix 2. Self-assessment Scales 38](#_Toc9757)

1. **Introduction and Rationale**

**1.1 Introduction**

Hypertension, also known as high blood pressure, is a major public health concern in China^1^. According to the latest statistics, approximately 27.9% of Chinese adults suffer from hypertension, which translates to over 330 million people. This alarming trend is largely attributed to rapid urbanization, unhealthy lifestyle choices, and an aging population. The burden of hypertension is not only limited to its high prevalence but also it's associated complications^2-4^, such as stroke, heart disease, and kidney failure, which pose significant challenges to the healthcare system.

In response to this growing epidemic, mobile health management has emerged as a promising approach to improve hypertension management and control^5, 6^. By leveraging mobile technology, such as smartphones and wearable devices, patients can easily monitor their blood pressure, track their medication adherence, and receive personalized health education and coaching^7, 8^. This trend is particularly relevant in China, where mobile phone penetration has reached over 98% and mobile health apps have gained widespread popularity^9^.

The clinical significance of mobile health management for hypertension lies in its potential to improve patient outcomes and reduce healthcare costs^10^. By empowering patients to take an active role in their own care, mobile health management can enhance medication adherence^7^, promote healthy lifestyle behaviors^11^, and facilitate timely interventions when necessary. Moreover, it can enable healthcare providers to remotely monitor patients' health status, identify high-risk individuals, and provide targeted interventions, thereby reducing the burden on the healthcare system^2^.

In summary, the utilization of mobile health management presents a hopeful development in the management of hypertension, particularly in China where hypertension is prevalent and mobile technology is extensively accessible. As healthcare providers, it is incumbent upon us to adopt this trend and capitalize on its potential to enhance patient outcomes and decrease healthcare expenses. In this study, we assessed the therapeutic efficacy of a novel multimodal digital transformation management (MDTM) model in the treatment of hypertension.

**1.2 Rationale**

We propose a model that integrates multimodal data to establish real-time, dynamic, accurate, and personalized hypertension health management to provide individualized treatment and risk stratification for hypertensive patients. WeChat, a leading social media platform in China, has emerged as a powerful tool for managing hypertension and promoting healthy lifestyles among the Chinese population^12^. With its user-friendly interface and extensive network of users, WeChat offers a unique advantage in delivering health-related information and support to individuals with hypertension. Our self-developed MDTM model, based on WeChat mini-program, features an integrated approach that includes visual and personalized model-based risk assessment, patient-centered, dynamic, real-time, personalized home telemonitoring, digital transformation of multimodal intervention for online health education and personalized primary prevention programs for cardiovascular disease, and chronic disease management programs. Experienced clinicians utilize the MDTM model to evaluate the patient's personal data and identify potential risk factors. Subsequently, they offer online medication management and medically validated non-pharmacological interventions, in accordance with Chinese hypertension management guidelines. The principal aim of the non-pharmacological interventions provided by the MDTM model is to mitigate the risk factors associated with hypertensive patients using health education and modifications to lifestyle, thereby enhancing health consciousness.

The health education program of the MDTM model includes multimodal interventions such as WeChat online interaction, health graphics, hypertension knowledge push, health talks, and video chats. The program aims to establish a favorable environment for hypertension patients, enabling them to fully comprehend the knowledge of hypertension health, increase awareness and lifestyle behaviors, apply health knowledge and lifestyle behaviors in practice, and accelerate self-management in hypertension. The system adopts different intervention methods according to personal information such as age, gender, interests, and living environment, allowing patients to learn health knowledge in a pleasant atmosphere and develop healthy behaviors. Lifestyle changes include adopting an appropriate diet (low salt, low fat, and low calorie), guidance on exercise patterns and timing, smoking and alcohol cessation, improved sleep patterns, psychological care, and weight loss strategies.

The hypertension management of the MDTM model aims to enhance self-monitoring awareness and medication adherence through continuous blood pressure self-monitoring, setting blood pressure control goals and follow-up management plans. Patients are encouraged to self-monitor their blood pressure at home every morning and actively upload their readings to the MDTM model. The application will categorize and visually display the patient's blood pressure to both the patient and healthcare provider, who will then set blood pressure control goals based on a week's worth of hypertension data.

As per the follow-up schedule, patients in the intervention group will be followed up online once a week and as an outpatient every three months. During these follow-up visits, healthcare practitioners will use the MDTM model to assess medication adherence, lifestyle changes, blood pressure levels, and adverse event outcomes. They will also provide personalized health education, lifestyle changes, and medication advice to patients in person or via WeChat.

**2. Study Design and Purpose**

**2.1 Study Design**

The study is designed to be a randomized, controlled trial conducted to test the therapeutic benefit of novel multimodal digital transforming management (MDTM) model with hypertension.

The randomized controlled trial design for mobile health management of hypertension involves the random allocation of participants into either a digital transformation of a multimodal intervention group or to usual care group. The intervention group receives mobile health management services, which include remote monitoring of blood pressure, medication reminders, lifestyle coaching, and personalized feedback. The usual care group receives usual care, which may include regular visits to a healthcare provider and self-monitoring of blood pressure.

The study will involve a pre-intervention baseline assessment of participants' blood pressure, medical history, lifestyle, and demographic information. Clinical outcome measure follow-up clinics were performed at 3 and 6 months. Participants will be followed up at regular intervals throughout the study period to assess changes in blood pressure, medication situation, and lifestyle behaviors. The primary outcome measure will be the change in systolic blood pressure from baseline to the end of the study period. The study will also include a process evaluation to assess the quality of life. Overall, the randomized controlled trial design for mobile health management of hypertension aims to evaluate the effectiveness and feasibility of using the MDTM model to improve blood pressure control and reduce cardiovascular risk among individuals with hypertension.

**2.2 Study Purpose**

**2.2.1 Primary Objective**

To assess the effectiveness of the MDTM model in lower blood pressure (BP) compared to standardized therapy among people with new onset hypertension 6 months after enrollment.

**2.2.2 Secondary Objectives**

To assess the effectiveness of the MDTM model in improving (a) lifestyle modification, (b) quality of life, (c) adherence to antihypertensive medication, and (d) level of daily activity, compared to optimal therapy among people with new onset hypertension at 6 months after enrollment.

**3. Study Population**

**3.1 Enrollment Population**

Our study population will be adult individuals between the ages of 20 and 80, who had a first diagnosis of essential hypertension ( BP measured at home three times on different days with a systolic BP > 140 mmHg or diastolic BP > 90 mmHg), had not taken antihypertensive medications before enrollment, utilized smartphones every day, and were considered appropriate to be managed with lifestyle modification for six months.

**3.2 Inclusion and exclusion criteria.**

| **Inclusion criteria** |
| --- |
| New onset hypertension |
| Patients had not taken antihypertensive medications before enrollment |
| Utilized smartphones every day |
| Local resident population |
| Signed consent form and agreed to receive 6-month intervention |
| Promised not to drop out due to the long term leave, going abroad or other reason during the next 6-month |
| Patients were considered appropriate to be managed with lifestyle modification for a period of six months. |
| **Exclusion criteria** |
| Secondary hypertension |
| Hypertension emergency |
| Secondary dyslipidemia patients (secondary to patients with type 2 diabetes not included) |
| Acute myocardial infarction or stroke within 3 months; |
| Depressed left ventricular systolic function (ejection fraction <30%) |
| Patients with chronic kidney disease (eGFR <60ml/min/1.73m2 or urine albumin/creatinine ratio≥30) |
| Pregnant women and nursing mothers |
| Malignant tumor |
| Dilated cardiomyopathy |
| Rheumatic heart disease |
| Cor pulmonale |
| Myocarditis or cardiomyopathy |
| Pregnant women and nursing mothers |
| With serious diseases, and life expectancy is less than one year |
| Dementia |
| Chronic kidney disease with proteinuria |
| Severe hepatic insufficiency |
| Free from orthostatic hypotension |
| Healthcare providers |
| Poor compliance to treatment |
| Lost to follow-up. |

**3.3 Informed Consent**

Subject Recruitment, Screening, and Enrollment for the MDTM Clinical Trial are conducted at Renmin Hospital of Wuhan University. We identify potentially eligible patients through the hospital's electronic system and outpatient clinics and schedule next-step recruitment counseling. We make telephone calls or provide on-site consultations in the outpatient clinic to all potential patients who meet the primary inclusion criteria. During the participant recruitment screening process, patients who are willing to participate in the trial will be asked to verbally allow the MDTM investigators to enter their information into the database for the next step of patient screening. Intended patients will be fully screened at the beginning of the screening visit to assess their eligibility for the trial. Patients who pass the screening visit will be considered eligible for the informed consent process. During this process, we will provide the eligible patient with a detailed verbal description of the study, including its risks, potential benefits, and requirements. We will also provide a paper copy of the informed consent form for the eligible patient to read, who will be given ample time to read and reflect on participation. If requested, individuals will be given additional time to consider participation, including rescheduling screening visits. Each individual will be allowed to ask questions before written consent is obtained until the individual can make a decision. When ready, participants will be asked to sign the consent form. We will then collect data on patients who provide informed consent, which will include information from the eligible patient's electronic health record and study procedures.

**3.4 Early Withdrawal of subjects**

| **Withdrawal criteria** |
| --- |
| Patient's medical record data failing to meet study requirements; |
| Patient refusal to be followed up; |
| Patients participating in other clinical studies； |
| Other major diseases that may affect life and health occur during the follow-up period; |
| Accidental occurrence during the patient's follow-up. |

**3.5 Replacement of participant**

Randomized subjects will not be replaced if discontinued.

**3.6 Early termination of the study**

This study may be temporarily suspended or terminated early if there is good and reasonable cause for such termination.

| **Suspended or terminated criteria** |
| --- |
| Determination of unexpected, significant or unacceptable risks to participants |
| Identify patients who may be at risk for an Serious Adverse Events(SAE) |
| Inadequate compliance with protocol requirements by the investigator |

**3.7 Interim analyses**

No interim analyses were planned or conducted.

**3.8** **Screening, Randomization, and Follow-up Flowchart**

**
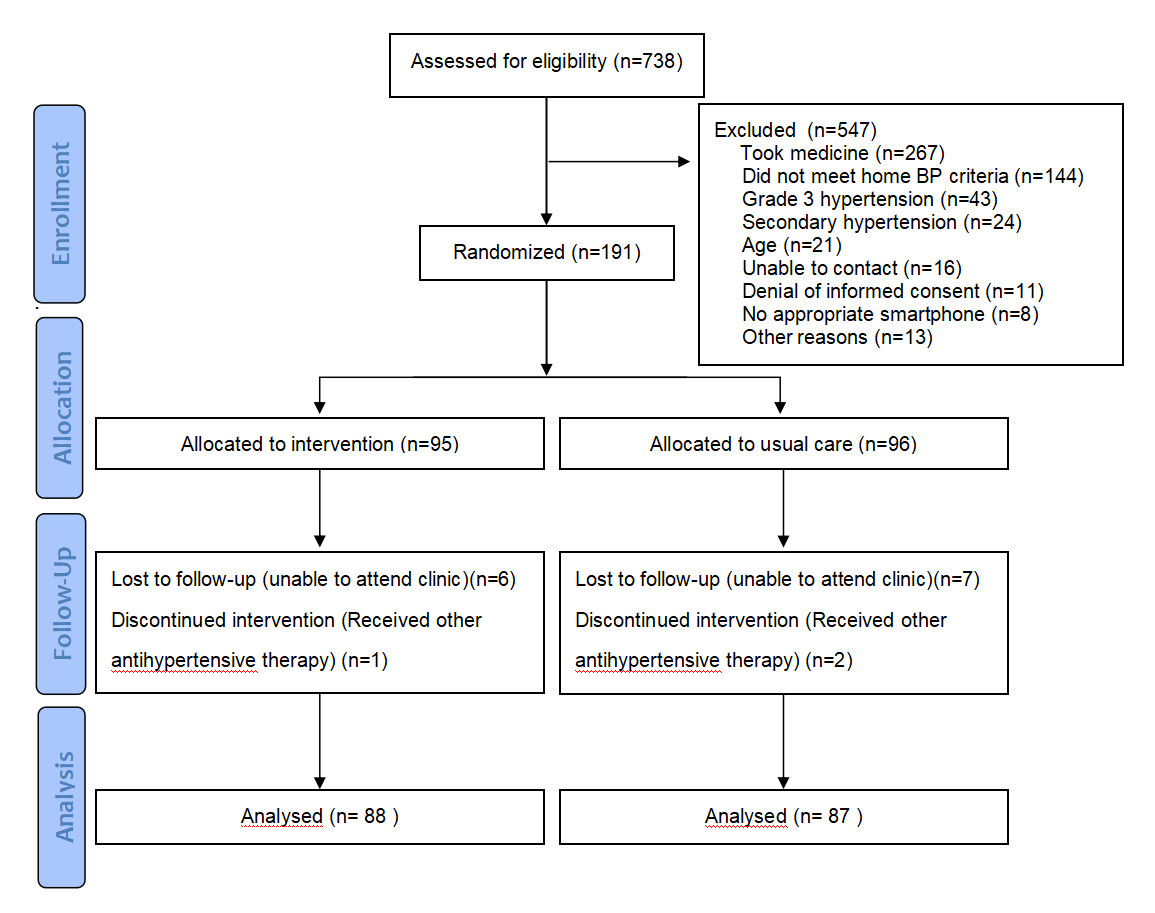
**

1. **Study Procedures**

The MDTM model is a digital transformation of a closed-loop healthcare system that is specifically designed to manage hypertension. This model integrates real-time self-monitoring of blood pressure, the establishment of optimized targets for blood pressure control, facilitated by the use of WeChat, which provides a user-friendly platform for patients and clinicians to access online health information through a visual interface. Additionally, the MDTM model implements a shared, personalized primary prevention program for cardiovascular disease and chronic disease management, which actively engages both patients and clinicians.

During the study, the patient's medical team allowed all the antihypertensive drugs simultaneously. The patient's medical team was allowed to continuously change the antihypertensive medication (including medication initiation, withdrawal, and / or dose change) throughout the study.

**4.1 Randomization**

The allocation sequence was generated by Yijun Wang via computer, while Jun Wang utilized an independent network-based group block randomization system to facilitate random assignment. Participants were subsequently enrolled by Shoupeng Duan, Zeyan Li, Xiaomeng Yang, and Lingpeng Song, and ultimately assigned to interventions by Yijun Wang and Jun Wang.

This study used block randomization with block sizes of 4 and 6 to ensure equal allocation.Participants were randomly assigned with equal probability to the multimodal intervention group (intervention group) or to usual care group (control group) on the basis of an independent web-based block randomization system. Randomisation was stratified by general practice with minimisation for sex, age (>60 or ≤60 years), BMI (＜30 or ≥30 kg/m^2^), baseline systolic BP (＜160 mm Hg vs ≥160 mm Hg) and presence or absence of diabetes and coronary artery disease. The patients in both trial groups received optimal-therapy, as recommended by experienced clinicians in cardiovascular medicine, which could contain a primary lifestyle modifications and standardized management for hypertension.

**4.2 Blinding**

Neither the participants, treating clinicians, nor the researchers were blinded.

**4.3 Intervention and Control**

**4.3.1 Intervention Strategy**

The purpose of the MDTM model is to digitally transform closed-loop management for hypertension, based on the Chinese hypertension management guidelines.The intervention will last 6 months, which included the following components : 1. real time BP self-monitoring and setting optimized targets for BP control; 2. building a digital mobile system to obtain health information; 3. personalized primary prevention program for cardiovascular disease, and chronic disease management programme ; 4. establishment of a multimodal healthcare platform to facilitate shared decision-making between patients and clinicians.

**(1). Real-time BP self-monitoring and setting optimized targets for hypertension management.**

The first step in developing a real-time BP self-monitoring system is to select an appropriate BP measurement device^8, 13-16^. This device should be accurate, reliable, and easy to use. Once the device is selected, the next step is to develop a mobile application that can display the BP readings in real-time. The MDTM model should also allow the user to set optimized targets for healthy life management based on their individual health status and medical history. WeChat Mini Program is a visualization tool that display a trends in previous BP measure how BP distribution has shifted over time for patients and clinical doctors.

**(2). Building a digital mobile system to obtain routine health information.**

To build a digital mobile system for personal evaluation, a comprehensive set of risk factors should be identified. These risk factors can include lifestyle factors such as smoking, alcohol consumption^17-19^, lifestyle modifications^20^, and daily activities^21^. The WeChat Mini Program is a visualization tool that displays online health information for patients and clinical doctors. Clinicians performed using baseline data and online health information to establish evaluate and systematize the presence of various risk factors and generate a personalized program of lifestyle modifications designed to manage hypertension.

**(3). Personalized primary prevention program for cardiovascular disease and chronic disease management program.**

To develop a personalized primary prevention program for cardiovascular disease, a thorough assessment of the user's medical history and risk factors should be conducted. This can be achieved through a combination of medical tests and questionnaires. Once the user's risk profile is established, a personalized prevention program can be developed that includes lifestyle modifications^22^, such as diet^23^ and exercise^21, 24, 25^, as well as use lifestyle modification approaches to manage sleep^26, 27^, anxiety^28^, and depression^28^. Similarly, a personalized chronic disease management program can be developed that includes regular monitoring of symptoms, medication management, and lifestyle modifications. In addition, clinician, who can then provide appropriate online health education according to patients’ individual circumstances and objective conditions^29^.

**(4). Establishment of a multimodal healthcare platform to facilitate shared decision-making between patients and clinicians.**

A digital platform, referred to as a multimodal healthcare platform, facilitates communication and collaboration between patients and clinicians in healthcare decision-making. The platform integrates diverse communication modes, including text, video, and voice, to enable patients and clinicians to connect in a manner that is most convenient for them. The platform would additionally grant entry to pertinent health data, encompassing daily blood pressure readings, lifestyle adjustments and advancement in the recommended regimen. This data would be presented in a comprehensible manner, enabling patients to make knowledgeable choices regarding their healthcare. Consequently, patients and clinicians can assess treatment alternatives and make decisions grounded on the most reliable evidence. A multimodal healthcare platform is intended to enhance shared decision-making between patients and clinicians, thereby aiming to enhance patient outcomes and satisfaction.

Online health education are summarized as popular science education to the each patient through multimodal interventions , including internet and online communication^30^, textual health education^31^, medical graphic narratives, animation-supported health education^32^. Online health education contains various kinds lessons ^33^on health management of hypertension education (eg, on reduce sodium intake and increase potassium intake^34^ through a balanced diet^35^. maintaining a healthy weight^25, 36, 37^, avoiding smoking, limiting alcohol consumption, increasing physical activity^21^, and managing sleep are also important lifestyle factors ) . Moreover, we provide tailored weekly hypertension health education programs that are culturally sensitive, age-appropriate, and consider other pertinent factors to ensure their contextual appropriateness and efficacy.

Together, the purpose of online health education is to make it easy for providing further health education and bridging the existing knowledge gap between clinicians and various populations. Program health education, lifestyle modifications and modifiable risk factors are fundamental elements in the context of online health education, consisting of how to choose an appropriate diet, guidance on exercise patterns and timing, explain the hazards of smoking and drinking, improved sleep patterns, and psychological care.

The multimodal intervention group comprises cardiologists who possess extensive expertise in their respective professional domains. They underwent standardized training to ensure the consistent provision of high-quality health education. We employed standardized health education materials to minimize variations among doctors. Additionally, regular quality control and feedback mechanisms are implemented to ensure the efficacy of the intervention. Involving patients in the digital transformation of health information is a crucial step as it empowers them to better comprehend and manage their health conditions. To guarantee the effectiveness of our intervention, we adopted several strategies: providing detailed guidance and support for patients to correctly utilize WeChat-based multimodal intervention, regularly monitoring patients' usage while offering feedback and suggestions, as well as conducting biweekly evaluations on effectiveness to monitor and enhance outcomes.

**4.3.2 Control group**

For the usual care group (control group), patients are required to self-record BP measurements every week and provide feedback to the clinical physician during outpatient follow-up, and the clinical physician adjusts the antihypertensive therapy based on the BP situation. The principles of low-dose initiation, long-acting medication, combination therapy, and individualization were used according to the Chinese hypertension management guidelines, which consisted of routine care for the prevention or treatment of hypertension by physicians. Moreover, all patients were provided with details on lifestyle modifications for the management of hypertension through the doctor's oral instructions oriented with the Chinese hypertension management guidelines.

**4.4 Study Duration**

Subjects will receive intervention for a minimum of 6 months.

**4.4.1 Study Outcome**

There will be two examinations for both groups, at baseline and 6 months (after 6-month intervention). Indicators including home and office BP differences after 6 months, home and office BP control rate, home and office BP levels, and quality-of-life scores (self-rating anxiety scale [SAS], the self-rating depression scale [SDS], and the Pittsburgh Sleep Quality Index [PSQI]) will be assessed.

All outcome assessments will be conducted at baseline and 6-month follow

up in the same way in the clinics for all patients, irrespective of their

assignment to intervention or control group. According to the follow-up schedule, patients in the digital transformation of the multimodal intervention group were followed up online once a week and as an outpatient conducted at 3, and 6 months.

**(1). Primary outcome**

The primary composite outcome was set as the difference in BP after 6 months, adjusted for baseline BP.

**(2). Secondary outcomes**

Major secondary outcomes were control rates of BP (BP ＜140/90 and＜130/80  mmHg)^38^, and the difference in quality-of-life scores after 6 months, contained SAS, SDS, and PSQI .

**4.4.2 Clinical Assessments**

**(1). Blood pressure measurement**

Eligible patients were taught how to employ automated electronic sphygmomanometers (Omron M10-IT; Omron Healthcare Europe, Hoofddorp, Netherlands) that were validated. All participants were scheduled to measure their own morning home BP in their right arm according to standard recommendations on every weekend. Eligible patients were scheduled to rest for at least five minutes before measurement and take a reading every minute between measurements in order to obtain accurate blood pressure measurements. A third measurement was administrated when the difference between the first 2 measurements was more than 5 mmHg for BP level. Analyses were carried out using the last two readings. Given the substantial body of previous research that has established a robust link between morning hypertension and the development of cardiovascular diseases, we have selected morning blood pressure as the primary endpoint of our study.

**(2). Quality-of-life scores**

We performed psychometric psychological-related measures including SAS, SDS, and PSQI. The main components of these four measures were calculated to indicate the level of negative affect and sleep quality.

**Self-rating anxiety scale (SAS)**

The SAS^40^ is a tool used to assess the level of anxiety in an individual. It consists of 20 questions, each with a score of 1 to 4, with 1 indicating "not at all" and 4 indicating "very much so". The total score is calculated by adding up the scores for all 20 questions, with a maximum score of 80. The SAS was used to rate normal (below 50), mild anxiety (50-59), moderate anxiety (60-69), and severe anxiety (70 or more) .

It is important to note that the SAS is not a diagnostic tool and should not be used as the sole basis for diagnosing anxiety disorders. It is recommended to seek professional help if you are experiencing symptoms of anxiety.

**Self-rating depression scale (SDS)**

The SDS^41^ is a tool used to assess the severity of depression in individuals. The SDS consists of 20 questions about the physical, emotional and psychological symptoms of depression. The score for each question ranges from 1 (rarely) to 4 (always), with higher scores indicating greater levels of depression. The total score is obtained by adding up the scores for each question, with a maximum possible score of 60. A higher score indicates a greater severity of depression. To obtain the standardized score, the raw score is multiplied by 1.25 and rounded to the nearest whole number. This standardized score is used to compare an individual's level of depression to that of a larger population. The maximum score is 60. A score of 53-62 indicates mild depression, 63-72 indicates moderate depression, and 72 indicates severe depression.

The SDS is not intended to be used as a diagnostic tool, but rather as a screening tool to identify individuals who may require further evaluation for depression. It is important to note that a high score on the self-rating depression scale does not necessarily indicate the presence of clinical depression, and a diagnosis should only be made by a qualified healthcare professional.

**Pittsburgh Sleep Quality Index (PSQI)**

The PSQI^43^ is a self-reported questionnaire that assesses sleep quality and disturbances over a one-month time interval. The questionnaire consists of 19 items that are grouped into seven components: subjective sleep quality, sleep latency, sleep duration, habitual sleep efficiency, sleep disturbances, use of sleeping medication, and daytime dysfunction. Each component is scored on a scale of 0 to 3, with 3 indicating the greatest severity of sleep disturbance. The seven component scores are then added together to yield a global score ranging from 0 to 21, with higher scores indicating poorer sleep quality. The global score is calculated by adding up the scores for each of the seven components, with a maximum possible score of 21. The PSQI score is classified into different levels based on the total score. A score of 0-5 indicates good sleep quality, a score of 6-10 indicates mild sleep quality, a score of 11-15 indicates poor sleep quality, and a score of 16 or higher indicates serious sleep quality.

**(3). Body Mass Index**

Body mass index (BMI) (calculated as weight in kilograms divided by height in meters squared) was calculated from self-reported height and weight. Non-obese BMI was defined as <30 Kg/m^2^ and obesity was defined as BMI of ≥ 30 Kg/m^2^.

**(4). Height Measurement**

The patient will remove shoes and wear light clothing; the patient stand in front of the height measuring instrument with feet together and heels, sit bones and back firmly against the instrument; the physician assistant will adjust the instrument so that it touches the top of the patient's head and press gently until it cannot go any lower; record the measurement, rounding to the nearest 0.1 cm.

**(5). Weight Measurement**

The patient will remove heavy clothing and wear light clothing; the patient will stand barefoot on the weight measuring device; the device will be adjusted so that it is level and stable and the measurement will begin without fluctuation; the patient will wait a few seconds until the number is stable and the measurement will be recorded; the physician's assistant will give the patient the necessary instructions, including the placement of the body's center of gravity and how to balance to ensure accurate measurement.

**4.4.3 Statistical Method**

All patients who completed the 6 months follow-up were included in the analysis

for the primary outcome and secondary outcome.

Normally distributed continuous variables are expressed as mean ± standard deviation (SD), and medians and interquartile ranges (IQRs) are otherwise presented.

Categorical variables were analyzed using the χ^2^ test or the Fisher exact test, while continuous variables were conducted to analyze with the t-test or Mann-Whitney test (as appropriate). The blood pressure values at various time points during the intervention were analyzed using repeated measures analysis of variance. To assess the intervention effect, the disparity between two groups was calculated by subtracting the difference in values at 6 months and baseline for the control group from that of the intervention group. Subsequently, an independent t-test was conducted to determine differences between pre- and post-intervention data within each group. A p-value below 0.05 indicates a statistically significant divergence in interventions observed among both groups. Additionally, we conducted exploratory analyses in predefined subgroups, including sex, age (>60 or ≤60 years), BMI (＜30 or ≥30 kg/m^2^), current smoking, current drinking, baseline systolic BP (＜160 mm Hg vs ≥160 mm Hg) and presence or absence of diabetes as well as coronary heart disease.

The study was not powered to show differences in the incidence of clinical events such as death, acute myocardial infraction, percutaneous coronary intervention , or drug type for monotherapy between multimodal intervention group and the usual care group, for which reason no statistical testing was performed regarding these variables.

**4.4.4 Sample Size Consideration**

The sample size estimation was according to the test of two independent proportions with a 2.5% Type I error rate, intracluster correlation coefficient of 0.01, and 80% retention rate. We aimed to have 90% power to detect a 20% absolute difference in BP control rate between the intervention and usual care group at the 6-month follow-up. We calculated that at least 80 evaluable patients would be required per group for the study to achieve this power.

**5.Data**

**5.1 Data Collection**

We will take certain confidentiality measures during data collection to ensure the security and confidentiality of patient data. We will use anonymous patient codes instead of the patient's real name, address and other personal information.

**5.2 Data Transmission**

Researchers ensure that data is also secure during transmission by using encryption technology, virtual private networks, and other means to secure data transmission.

**5.3 Data Processing**

We will use appropriate data processing measures to ensure the security and confidentiality of the data. For example, we will limit access authorizations and employ password protection and other security measures to protect data from unauthorized access, use or disclosure.

**5.4 Data Storage**

We will retain data for a minimum of one year after the completion of the study for future reference or data sharing purposes. During this period, we will take the necessary confidentiality measures to ensure the security and confidentiality of the data.

**5.5 Data Sharing**

We will share data only as necessary and only with authorized researchers and regulatory agencies. We will take the necessary steps to ensure the security and confidentiality of the data.

**5.6 Data Retention Period**

We will retain data after the study is completed for future reference or data-sharing purposes. During this time, we will take the necessary steps to ensure the security and confidentiality of the data.

**5.7 Cancellation of Participation**

We will allow patients to cancel their participation in the study at any time and, upon cancellation, we will stop collecting their data and will not use or share any of their data.

**5.8 Researcher Confidentiality**

All researchers involved in the data must sign confidentiality agreements to ensure that the identity of participants and research data are not disclosed. Researchers must also take steps to protect their personal accounts and passwords to ensure that unauthorized persons cannot access research data.

**6. Safety**

We take the following steps to ensure the safety of participants and minimize the occurrence of adverse events. We will strictly abide by national and international ethical norms and regulations to ensure that all research practices meet ethical and legal standards.

**6.1 Adverse Events**

**6.1.1 Definition**

**Adverse Events (AE) ：**

An adverse event (AE) is any symptom, sign, illness, or experience that develops or worsens in severity during the course of the study. Intercurrent illnesses or injuries should be regarded as adverse events. Common hypertensive adverse events are as follows: Cough, Stomach discomfort, Edema, Dizziness, Other.

**Serious Adverse Events (SAE)：**

Serious adverse events refer to any adverse events occurring above a certain level/frequency among participants. Serious adverse events meet at least one of the following conditions:

1. **. Life-threatening**

The participant's life was threatened and required urgent medical intervention, e.g., cardiopulmonary arrest, severe allergic reaction, etc.

Adverse event requiring hospitalization: The participant required hospitalization for further diagnosis and treatment, e.g., severe hypertensive crisis.

**(2). Residual sequelae or lasting effects**

Participants experienced adverse events resulting in residual sequelae or lasting effects, such as permanent vision loss, permanent kidney damage, etc.

**(3). Other serious adverse events**

Other adverse events that could lead to threatening or serious effects on the participant, such as suicidal behavior, accidents, etc.

**6.1.2 Monitoring**

We will monitor participants for adverse events, including drug-related adverse reactions, lifestyle intervention-related problems, etc. All adverse events will be recorded and reported, and investigators will take the necessary interventions as needed.

**6.1.3 Solution**

When an adverse event occurs in a clinical study, resolution should be based on the type and severity of the adverse event. The following are solutions for adverse events that may occur in this study:

**(1).Medication regimen adjustment**

For medication-related adverse events, investigators should consult with their physicians and consider whether medication dose adjustments, medication changes, or discontinuation of medications are necessary.

**(2). Lifestyle adjustment**

For lifestyle intervention-related adverse events, investigators should consult with the patient and consider the need to adjust the lifestyle intervention program, such as adjusting the diet plan, exercise intensity, etc.

**(3). Emergency management**

For serious adverse events, investigators should take immediate emergency management measures, such as calling an emergency vehicle, stopping medication, etc.

1. **. Monitoring and recording**

Researchers should regularly monitor and record the occurrence of adverse events, including information on the type, time of occurrence, duration, and severity of the event.

**(5). Reporting and notification**

For all adverse events that occur, investigators should report and notify relevant parties, such as patients, regulatory agencies, and study committees, in a timely manner.

**(6). Follow-up**

For patients who have an adverse event, investigators should conduct follow-up visits to understand the follow-up of the event and take further management measures as needed.

**6.2 Safety of Data Collection**

We will collect safety data from participants, including vital signs, laboratory tests, etc. These data will be recorded and analyzed to ensure the health and safety of the participants.

**6.3 Safety of WeChat Data**

In conducting clinical research and collecting and processing patient personal data, this study will strictly adhere to privacy protection procedures for clinical research, data protection legislation, and ethical standards. Robust safeguarding measures will be implemented to ensure the security of participants' personal data, preventing any loss or theft, unauthorized access, misuse or disclosure, improper duplication, transmission, modification or tampering, improper retention or destruction.

During centralized screening in this study, individual informed consent requires a separate space after obtaining group informed consent. This arrangement ensures that participants can freely ask questions and receive answers without hesitation.

Throughout the process of this clinical research project, all forms such as scale collection records should be identified with unique identification numbers that comply with the regulations of this specific project. Personal information of participants such as names and genders should not be included. When staff members communicate publicly about the project details, they should use identification numbers instead of names.

Any documents or materials containing personal information of the participants should be securely stored in a designated location, promptly organized, and archived. Only authorized personnel are permitted to write or access these records, which should not be placed in high-traffic areas or open spaces. Unauthorized individuals must not handle any medical records related to the research. Research materials should be stored in an archive managed by a dedicated person, and files should be promptly locked after archiving. Borrowing of these materials is only allowed for authorized personnel and must be registered accordingly. For research centers that integrate research medical records with electronic medical records, it is essential to adhere to the "Medical Institution Medical Records Management Regulations" while recognizing that medical records used as research materials need rigorous and appropriate handling to prevent privacy breaches. Therefore, medical institutions should establish a robust system for utilizing clinical research medical records.

Upon completion of this clinical research and database locking, patient personal information will be anonymized as required so that individual identities cannot be identified within the database or final report. Access to anonymized data is limited solely to the principal investigator and their authorized data analysts. After completing thorough data analysis, scientific research papers will commence preparation followed by revisions, submission, and publication involving all authors. When publishing research findings, emphasis should be placed on reporting group aggregate data rather than individual participant data. In cases where individual special circumstances are described, any personally identifiable information about participants must not appear but can instead be replaced with coding.

**6.4 Study Discontinuation Rules**

We will set discontinuation rules in the study so that the study will be suspended or terminated when a serious adverse event or safety issue occurs. These rules will ensure that the health and safety of participants is the highest priority.

**6.5 Safety Oversight Committee**

We will establish a safety oversight committee to oversee the safety of the study. This committee, composed of professional physicians and experts, will monitor all safety data and make recommendations and interventions as necessary.

**7. Study Monitoring**

**7.1 Clinical Monitoring**

Researchers will conduct on-site monitoring at each outpatient follow-up visit. Clinical site monitoring is conducted to ensure that the rights and well-being of human subjects are protected, that the reported trial data are accurate, complete, and verifiable, and that the conduct of the trial is in compliance with the currently approved protocol/amendment(s) and with applicable regulatory requirement(s).

Also, the study team will assess and follow up on the subject's adherence. This will include:

**7.1.1 Medication Reminders and Instructions**

Researchers will monitor participants' medication taking, including the type, dose, frequency and duration of medication. Researchers will contact participants regularly to ensure they are following their medication regimen and to answer any medication-related questions they may have.

**7.1.2 Lifestyle Intervention**

Researchers will monitor participants' lifestyle behavior changes^44^, including smoking cessation, alcohol cessation, diet, exercise and sleep. Participants will be contacted regularly by telephone or face-to-face to ensure compliance with the intervention plan and to provide support and advice as needed.

**7.2 Study Process Supervision**

Researchers will randomly sample participants' data and check data accuracy and completeness. Researchers will also assess the performance of study personnel and provide support and guidance as needed.

**7.3 Adverse Event Monitoring**

Researchers will monitor participants for adverse events, including drug-related adverse reactions, lifestyle intervention-related problems, etc. All adverse events will be recorded and reported, and the investigator will take necessary interventions as needed.

**8. Ethical Considerations**

This protocol will be submitted to the Clinical Research Institutional Review Board (IRB) of Renmin Hospital of Wuhan University for formal approval of the study's conduct, in compliance with local laws. The IRB will communicate its decision regarding the study's conduct in writing to the investigator. All study participants will receive sufficient information about the study and will make an informed decision to participate by signing an informed consent form. This consent form, along with the protocol, will be submitted for IRB review and approval. Prior to any study procedures, formal consent must be obtained from the subject using the IRB-approved consent form, signed by both the subject and the designated investigator. The subject's informed consent form is provided in Appendix 1.

**9. Appendix**

**Appendix** **1. Informed Consent**

**Informed Consent**

**Title:**

Efficacy of Digital Transformation of Multimodal Healthcare System in the management of New-onset Hypertension : A MDTM Randomized Clinical Trial.

**Informed Consent • Informed Notice page**

**Dear participant,**

We would like to extend an invitation for you to take part in the clinical trial focused on the management of hypertension patients through the Digital Transformation of Multimodal Healthcare System. This study has undergone a thorough review and has been approved by the Ethics Committee of Renmin Hospital of Wuhan University.

Before making a decision about participating in this clinical study, we kindly ask you to carefully read the following information. This will help you understand the study's purpose, duration, requirements, and potential benefits, risks, and discomforts associated with participation. Please note that your participation is voluntary, and you have the right to choose whether to participate or not.

You may discuss this study and the information provided with your family, friends, doctor, or other trusted individuals. The study doctor will explain this information to you, and if you have any questions, they will be happy to answer them. If you decide to participate, you will be asked to sign an informed consent form (at the end of this document) before any study-related procedures are performed.

**Introduction**

Hypertension, commonly known as high blood pressure, is a significant public health concern in China. Recent statistics reveal that over 330 million people, or 27.9% of Chinese adults, suffer from hypertension. This trend is mainly attributed to unhealthy lifestyle choices, rapid urbanization, and an aging population. The burden of hypertension is not only limited to its high prevalence but also its associated complications, such as heart disease, stroke, and kidney failure, which pose significant challenges to the healthcare system. In response to this growing epidemic, mobile health management has emerged as a promising approach to improve hypertension management and control. By utilizing mobile technology, such as smartphones and wearable devices, patients can easily monitor their blood pressure, track their medication adherence, and receive personalized health education and coaching. This trend is particularly relevant in China, where mobile phone penetration has reached over 98%, and mobile health apps have gained widespread popularity. The clinical significance of mobile health management for hypertension lies in its potential to enhance patient outcomes and reduce healthcare costs. By empowering patients to take an active role in their own care, mobile health management can improve medication adherence, promote healthy lifestyle behaviors, and facilitate timely interventions when necessary. Furthermore, it can enable healthcare providers to remotely monitor patients' health status, identify high-risk individuals, and provide targeted interventions, thereby reducing the burden on the healthcare system.

In summary, the utilization of mobile health management presents a promising trend in the management of hypertension, particularly in China where hypertension is prevalent and mobile technology is readily accessible. As healthcare providers, it is incumbent upon us to adopt this trend and exploit its potential to enhance patient outcomes and decrease healthcare expenses. This study examines the therapeutic efficacy of a novel multimodal digital transformation management (MDTM) model in the treatment of hypertension.

**Research Purpose：**

**Primary Objective：**

To assess the effectiveness of multimodal digital transformation management model (MDTM model) in blood pressure (BP) compared to usual care among people with new onset hypertension 6 months after enrollment.

**Secondary Objectives:**

To assess the effectiveness of MDTM model in improving (a) lifestyle modification, (b) quality of life, (c) adherence to antihypertensive medication, and (d) level of daily activity, compared to optimal therapy among people with new onset hypertension at 6 months after enrollment.

**Inclusion and exclusion criteria.**

| **Inclusion criteria** |
| --- |
| New onset hypertension |
| Patients had not taken antihypertensive medications before enrollment |
| Utilized smartphones every day |
| Local resident population |
| Signed consent form and agreed to receive 6-month intervention |
| Promised not to drop out due to the long term leave, going abroad or other reason during the next 6-month |
| Patients were considered appropriate to be managed with lifestyle modification for a period of six months. |
| **Exclusion criteria** |
| Secondary hypertension |
| Hypertension emergency |
| Secondary dyslipidemia patients (secondary to patients with type 2 diabetes not included) |
| Acute myocardial infarction or stroke within 3 months; |
| Depressed left ventricular systolic function (ejection fraction <30%) |
| Patients with chronic kidney disease (eGFR <60ml/min/1.73m2 or urine albumin/creatinine ratio≥30) |
| Pregnant women and nursing mothers |
| Malignant tumor |
| Dilated cardiomyopathy |
| Rheumatic heart disease |
| Cor pulmonale |
| Myocarditis or cardiomyopathy |
| Pregnant women and nursing mothers |
| With serious diseases, and life expectancy is less than one year |
| Dementia |
| Chronic kidney disease with proteinuria |
| Severe hepatic insufficiency |
| Free from orthostatic hypotension |
| Healthcare providers |
| Poor compliance to treatment |
| Lost to follow-up. |

**How many people will participate in this study?**

The plan is to recruit 160 subjects in this study at our institution.

**Study Procedure**

(1) Before you are selected for the study, the research team will collect the following information and test results to determine whether you are eligible to participate in the study.

• Medical history: The study doctor will ask you some questions to understand any current or previous disease.

• Demographic information: The study doctor will collect information about you personally, such as your date of birth and ethnic background.

• Height and weight: Researchers will measure your height and weight.

• Vital signs: The study doctor will measure your blood pressure, heart rate and respiratory rate.

(2) If the above information and test results meet the requirements, the researchers will confirm that you have been included in this study, and you will be randomly assigned to the digital transformation of multimodal intervention group or usual care group for a total of 6 months of intervention and follow-up. During this period, researchers will observe the actual effects of your intervention use. To ensure that the effect of digital transformation of multimodal intervention group can be accurately recorded and evaluated, you need to cooperate with the following procedures during this period:

• Measure blood pressure regularly and send it to the doctor through digital transformation.

(3) When you have completed all follow-up visits, it is generally considered that you have completed the entire study. Please carefully consider the impact of the above tests and visits on your daily work and family life. At the same time, please consider the transportation arrangements for each visit, whether you need to make an appointment for the test, and whether some tests will cause discomfort. If you have any questions about the research tests or procedures, please ask the study doctor. Please reserve enough time to receive follow-up calls from researchers (if applicable). Please ensure that you inform your doctor or hospital of any changes in your health status in a timely manner.

**How long will this study last?**

This clinical trial will be conducted over a period of 6 months, during which you will be required to attend outpatient follow-up visits at 3 months, and 6 months. You have the right to withdraw from the study at any time without fear of discrimination or retaliation, and your decision to withdraw will not affect your medical treatment or rights. Your clinician or researcher may also suspend your participation in the study at any time if it is deemed to be in your best interest (the reason for possible termination of the trial will be explained to you). If you choose to participate in this study, we kindly request that you commit to completing the entire research process. In the event that you withdraw from the study for any reason, a relevant examination may be conducted to ensure your safety.

**1. Risks and/or discomforts of participating in this study**

There are no risks associated with participating in this study. However, there may be information security risks. We will do our best to protect the information you provide from being disclosed. Some of the questions we ask you in this study may make you feel uncomfortable, and you have the right to refuse to answer such questions. Additionally, you can take a break at any time during the study. At any point during the study, you can choose to withdraw from the study.

**2. Benefits of participating in the study**

If you agree to participate in this study, you may potentially receive direct medical benefits. Specifically, the digital transformation of multimodal intervention may help improve your BP control. However, we cannot guarantee this outcome. We hope that the information we obtain from your participation in this study will help provide more information for the diagnosis and treatment of hypertension in the future.

**3. Alternative treatment options if not participating in the study**

This study will not provide any other treatment options. Your diagnosis and treatment will be determined by the research doctor based on your condition, and you can continue with your regular treatment plan.

The use of research results and confidentiality of personal information: In this study, your personal information will be collected for statistical and analytical purposes. You will have the opportunity to learn about the research results. You can ask your research doctor for the results and ask for an explanation. The results of this study may also be published in journals or presented at conferences, but they will not contain any information that could identify you.

To ensure privacy, records or samples published for research purposes will not include your name or any other identifying information. Instead, your information will only be identified by a code. Only the research doctor and authorized personnel can link this code to your name through a list, which will be securely stored at the research center.

In order to ensure that the research is conducted in accordance with regulations, the applicant, ethics review committee, and government regulatory agencies may access your information when necessary. They are bound by confidentiality obligations and will not violate your privacy.

You have the right to control the use and disclosure of your personal information. You can request to view your medical information at any time, as permitted by national law. You have the right.

**Research-related updates**

During the course of the study, if there are any changes to the study protocol or application, your research doctor will immediately inform you and discuss with you whether you wish to continue participating in the study. If you decide not to continue, your medical treatment and rights will not be affected. If you choose to remain in the study, your research doctor may ask you to sign a new informed consent form.

**Study costs, compensation, and damages**

If you participate in this study, you will not be required to pay any additional fees. You will only be responsible for the costs associated with the relevant medical examinations for your underlying condition. You will not receive any financial compensation for your participation in the study. However, during the follow-up process, you will receive health guidance from our professional medical staff. If you suffer any harm as a result of participating in the study, you will receive professional treatment provided by the department of cardiology of Renmin Hospital of Wuhan University, and will be compensated in accordance with the law.

**Rights and Responsibilities of Participants**

**1. Rights**

Throughout the entire research process, your participation is voluntary. If you decide not to participate in this study, it will not affect any other treatment you may receive. If you choose to participate, you will be asked to sign this informed consent form. You have the right to withdraw from the study at any time without discrimination or unfair treatment, and your medical treatment and rights will not be affected.

**2. Responsibilities**

As a participant in this study, please abide by the following agreements:

• Return to the hospital for scheduled visits on time.

• You can inform your research doctor at any time if you wish to terminate the study.

• Provide truthful information about your medical history and current physical condition.

• Follow the instructions of the research staff.

• Inform the research doctor of any discomfort you experience during the study.

• Any experimental treatment may pose a risk to you or your fetus, so you and your partner should avoid any activities that may lead to pregnancy during the study. If you become pregnant during the study, please inform your research doctor immediately.

**3. Contact Information**

If you have any questions related to this study, please contact the researcher at 18643268984.

If you have any questions regarding your rights and interests, or if you wish to report any difficulties, dissatisfaction, or concerns during your participation in this study, or if you wish to provide feedback or suggestions related to this study, please contact the Ethics Committee of Renmin Hospital of Wuhan University at 027-88041911-81353.

**Informed Consent Form • Consent Signature Page**

**Participant Declaration:**

I have been given information about the study's background, purpose, methodology, potential risks, and benefits. I have had ample time and opportunity to ask questions, and I am content with the responses I have received. Additionally, I have been made aware of who to contact if I have any inquiries, concerns, recommendations, or would like to offer additional information or support for the study.

I acknowledge that my participation in this study is entirely voluntary, and I affirm that I have been given ample time to carefully consider and willingly consent to take part. I retain the right to withdraw from the study at any point without fear of any negative consequences or repercussions on my medical treatment or personal rights. Additionally, I have been assured that the researchers have not employed any deceitful tactics, coercion, or undue pressure to compel my participation in the study.

I acknowledge that in the event of my condition deteriorating, or if I encounter severe adverse reactions, or if my research doctor deems that my continued participation in the study is not in my best interest, he/she may withdraw me from the study. Additionally, the sponsor or regulatory agency may terminate the study during the research period without my consent. In such an event, my doctor will inform me promptly, and my research doctor will discuss alternative options with me.

I have carefully reviewed and understood the contents of this informed consent form, and I willingly consent to participate in this study. I acknowledge that I will be provided with a copy of the original informed consent form, which will include both my and the researcher's signature and the date of signing.

Participant Signature: Date:

Contact Phone Number:

Legal Representative Signature: Date:

Contact Phone Number:

(Note: If the participant has no legal capacity or limited legal capacity, such as inclusion of vulnerable groups with mental disorders/unconsciousness, the legal representative needs to sign at the following legal representative signature)

Fair Witness Signature: Date:

Contact Phone Number:

(Note: Only when it is possible to include participants with decision-making capacity but unable to read the text, such as illiteracy, visual impairment, a fair witness signature is required. The researcher should keep video materials as proof of informed consent when the witness is informed.)

Researcher Declaration:

I have accurately informed the participant of this document, and he/she has read this informed consent form accurately and confirmed that the participant had the opportunity to ask questions and voluntarily agreed. I have given him/her a signed original of the informed consent form.

Researcher Signature: Date:

Contact Phone Number:

**Appendix** **2. Self-assessment Scales**

**Self-rating anxiety scale (SAS)**

The SAS is a tool used to assess the level of anxiety in an individual. It consists of 20 questions, each with a score of 1 to 4, with 1 indicating "not at all" and 4 indicating "very much so". The total score is calculated by adding up the scores for all 20 questions, with a maximum score of 80. The SAS was used to rate normal (below 50), mild anxiety (50-59), moderate anxiety (60-69), and severe anxiety (70 or more) .

It is important to note that the SAS is not a diagnostic tool and should not be used as the sole basis for diagnosing anxiety disorders. It is recommended to seek professional help if you are experiencing symptoms of anxiety.

| Name: Sex: Medical Number: Assessor: | | | | |
| --- | --- | --- | --- | --- |
| Actual Feeling | Rarely | Sometimes | Often | Very Often |
| 1. l feel more nervous and anxious than usual. | 1 | 2 | 3 | 4 |
| 2. l feel afraid for no reason at all. | 1 | 2 | 3 | 4 |
| 3. l get upset easily or feel panicky. | 1 | 2 | 3 | 4 |
| 4. l feel like l'm falling apart and going to pieces. | 1 | 2 | 3 | 4 |
| *5. l feel that everything is all right and nothing bad will happen. | 4 | 3 | 2 | 1 |
| 6. My arms and legs shake and tremble. | 1 | 2 | 3 | 4 |
| 7. l am bothered by headaches neck and back pain. | 1 | 2 | 3 | 4 |
| 8. l feel weak and get tired easily. | 1 | 2 | 3 | 4 |
| *9. l feel calm and can sit still easily. | 4 | 3 | 2 | 1 |
| 10. l can feel my heart beating fast. | 1 | 2 | 3 | 4 |
| 11. l am bothered by dizzy spells. | 1 | 2 | 3 | 4 |
| 12. l have fainting spells or feel like it. | 1 | 2 | 3 | 4 |
| *13. l can breathe in and out easily. | 4 | 3 | 2 | 1 |
| 14. l get feelings of numbness and tingling in my fingers & toes. | 1 | 2 | 3 | 4 |
| 15. l am bothered by stomach aches or indigestion. | 1 | 2 | 3 | 4 |
| 16. l have to empty my bladder often. | 1 | 2 | 3 | 4 |
| *17. My hands are usually dry and warm. | 4 | 3 | 2 | 1 |
| 18. My face gets hot and blushes. | 1 | 2 | 3 | 4 |
| *19. l fall asleep easily and get a good night's rest. | 4 | 3 | 2 | 1 |
| 20. l have nightmares. | 1 | 2 | 3 | 4 |
| Total |  | | | |

Explanation:

Never or rarely: None or a little of the time (no more than one day in the past week).

Sometimes: Some of the time (1-2 days in the past week)

Often: Good part of the time (3-4 days in the past week).

Very often: Most or all of the time (5-7 days in the past week).

Scoring instructions:

Add up the scores of 20 questions to get the rough score X, multiply X by 1.25 (take an integer), get the standard score Y.

Scoring reference:

| SAS standard score Y | Anxiety Level |
| --- | --- |
| 50-59 | Moderate |
| 60-70 | Severe |
| >70 | Extreme |

**Self-rating depression scale (SDS)**

The SDS is a tool used to assess the severity of depression in individuals. The SDS consists of 20 questions about the physical, emotional and psychological symptoms of depression. The score for each question ranges from 1 (rarely) to 4 (always), with higher scores indicating greater levels of depression. The total score is obtained by adding up the scores for each question, with a maximum possible score of 60. A higher score indicates a greater severity of depression. To obtain the standardized score, the raw score is multiplied by 1.25 and rounded to the nearest whole number. This standardized score is used to compare an individual's level of depression to that of a larger population. The maximum score is 60. A score of 53-62 indicates mild depression, 63-72 indicates moderate depression, and 72 indicates severe depression.

The SDS is not intended to be used as a diagnostic tool, but rather as a screening tool to identify individuals who may require further evaluation for depression. It is important to note that a high score on the self-rating depression scale does not necessarily indicate the presence of clinical depression, and a diagnosis should only be made by a qualified healthcare professional.

| Name: Sex: Medical Number: Assessor: | | | | |
| --- | --- | --- | --- | --- |
| Actual Feeling | Rarely | Sometimes | Often | Very Often |
| 1. l feel down-hearted and blue. | 1 | 2 | 3 | 4 |
| *2. Morning is when I feel the best. | 4 | 3 | 2 | 1 |
| 3. l have crying spells or feel like it. | 1 | 2 | 3 | 4 |
| 4. l have trouble sleeping at night. | 1 | 2 | 3 | 4 |
| *5. l eat as much as l used to. | 4 | 3 | 2 | 1 |
| *6. l still enjoy sex. | 4 | 3 | 2 | 1 |
| 7. l notice that l am losing weight. | 1 | 2 | 3 | 4 |
| 8. l have trouble with constipation. | 1 | 2 | 3 | 4 |
| 9. My heart beats faster than usual. | 1 | 2 | 3 | 4 |
| 10. l get tired for no reason. | 1 | 2 | 3 | 4 |
| *11. My mind is as clear as it used to be. | 4 | 3 | 2 | 1 |
| *12. l find it easy to do the things l used to. | 4 | 3 | 2 | 1 |
| 13. l am restless and can't keep still. | 1 | 2 | 3 | 4 |
| *14. l feel hopeful about the future. | 4 | 3 | 2 | 1 |
| 15. l am more irritable than usual. | 1 | 2 | 3 | 4 |
| *16. l find it easy to make decisions. | 4 | 3 | 2 | 1 |
| *17. l feel that l am useful and needed. | 4 | 3 | 2 | 1 |
| *18. My life is pretty full. | 4 | 3 | 2 | 1 |
| 19. l feel that others would be better off if l were dead. | 1 | 2 | 3 | 4 |
| *20. l still enjoy the things l used to do. | 4 | 3 | 2 | 1 |
| Total |  | | | |

Explanation:

Never or rarely: None or a little of the time (no more than one day in the past week).

Sometimes: Some of the time (1-2 days in the past week)

Often: Good part of the time (3-4 days in the past week).

Very often: Most or all of the time (5-7 days in the past week).

Scoring instructions:

Add up the scores of 20 questions to get the rough score X, multiply X by 1.25 (take an integer), get the standard score Y.

Scoring reference:

| SDS standard score Y | Anxiety Level |
| --- | --- |
| 53-62 | Moderate |
| 63-72 | Severe |
| >72 | Extreme |

**Pittsburgh Sleep Quality Index (PSQI)**

The PSQI is a self-reported questionnaire that assesses sleep quality and disturbances over a one-month time interval. The questionnaire consists of 19 items that are grouped into seven components: subjective sleep quality, sleep latency, sleep duration, habitual sleep efficiency, sleep disturbances, use of sleeping medication, and daytime dysfunction. Each component is scored on a scale of 0 to 3, with 3 indicating the greatest severity of sleep disturbance. The seven component scores are then added together to yield a global score ranging from 0 to 21, with higher scores indicating poorer sleep quality. The global score is calculated by adding up the scores for each of the seven components, with a maximum possible score of 21. The PSQI score is classified into different levels based on the total score. A score of 0-5 indicates good sleep quality, a score of 6-10 indicates mild sleep quality, a score of 11-15 indicates poor sleep quality, a score of 16 or higher indicates indicates serious sleep quality.

| Name: Sex: Medical Number: Assessor: |
| --- |
| 1. During the past month, when have you usually gone to bed at night?   USUAL BED TIME |
| 1. During the past month, how long (in minutes) has it usually taken you to fall asleep each night?   NUMBER OF MINUTES |
| 1. During the past month, when have you usually gotten up in the morning?   USUAL GETTING UP TIME |
| 1. During the past month, how many hours of actual sleep did you get at night? (This may be different than the number of hours you spend in bed.)   HOURS OF SLEEP PER NIGHT |
| 5. During the past month, have you had trouble sleeping due to the following conditions? Please check the one best response from ①②③④: |
| 1. Cannot get to sleep within 30 minutes.    1. None ②Less than once a week ③Once or twice a week ④Three or more times a week |
| 1. Wake up in the middle of the night or early morning.    1. None ②Less than once a week ③Once or twice a week ④Three or more times a week |
| 1. Have to get up to use the bathroom.    1. None ②Less than once a week ③Once or twice a week ④Three or more times a week |
| 1. Cannot breathe comfortably.    1. None ②Less than once a week ③Once or twice a week ④Three or more times a week |
| 1. Cough or snore loudly.    1. None ②Less than once a week ③Once or twice a week ④Three or more times a week |
| 1. Feel too cold.    1. None ②Less than once a week ③Once or twice a week ④Three or more times a week |
| 1. Feel too hot.    1. None ②Less than once a week ③Once or twice a week ④Three or more times a week |
| 1. Had bad dreams.    1. None ②Less than once a week ③Once or twice a week ④Three or more times a week |
| 1. Have pain.    1. None ②Less than once a week ③Once or twice a week ④Three or more times a week |
| 1. Other reason(s), please describe   How often during the past month have you had trouble sleeping because of this?   - 1. None ②Less than once a week ③Once or twice a week ④Three or more times a week |
| 6. During the past month, how would you rate your sleep quality overall?  ① Very good ②Fairly good ③Fairly bad ④Very bad |
| 7. During the past month, how often have you taken medicine (Prescribed or "over the counter"') to help you  Sleep?   1. None ②Less than once a week ③Once or twice a week ④Three or more times a week |
| 8. During the past month, how often have you had trouble staying awake while driving, eating meals, or engaging in social activity?  ① None ②Less than once a week ③Once or twice a week ④Three or more times a week |
| 9. During the past month, how much of a problem has it been for you to keep up enough enthusiasm to get things done?  ①No problem at all ②Only a very slight problem ③Somewhat of a problem ④A very big problem |

**Scoring instructions:**

**A. Sleep Quality**

Question 6: ① О points, ② 1 point, ③ 2 points, ④ 3 points

**B. Time to Fall Asleep**

1. Question 2: "≤15" gets 0 points, "16~30" gets 1 point, "31~60" gets 2 points, "≥60" gets 3 points.

2. Question 5a: ① О points, ② 1 point, ③ 2 points, ④ 3 points

3. Accumulate the scoring of questions 2 and 5a: if the cumulative score is "O", О points will be awarded, "1~2" will be assigned 1 point, "3~4" will be assigned 2 points, and "5~6" will be assigned 3 points.

**C. Sleep Time**

Question 4: ">7 hours" gets 0 points, "6~7 hours" gets 1 point, "5~6 hours" gets 2 points, "<5 hours" gets 3 points.

**D. sleep efficiency**

1. Time in bed = topic 3 (time to get up) - topic 1 (time to go to bed)

2. Sleep efficiency = Question 4 (sleep time) / time in bed × 100%

3. Sleep efficiency: ">85%" counts О points, "75~84%" counts 1 point, "65~74%" counts 2 points, "<65%" counts 3 points

**E. sleep disturbance**

1. Items 5b to 5j: ① 0 points, ② 1 point, ③ 2 points, ④ 3 points

2. Accumulate the scoring of questions 5b to 5j:

if the cumulative score is "O", 0 points are awarded, "1~9" is assigned 1 point, "10~18" is assigned 2 points, and "19~27" is assigned 3 points

**F. Hypnotic drugs**

Question 7: ① О points, ② 1 point, ③ 2 points, ④ 3 points

**G. Daytime dysfunction**

1. Question 8: ① О points, ② 1 point, ③ 2 points, ④ 3 points

2. Question 9: "No" is 0 points, "Occasionally" is 1 point, "Sometimes is" is 2 points, "Often" is 3 points

3. Add up the scores for questions 8 and 9:

If the cumulative score is "0", 0 points are awarded, "1~2" is assigned 1 point, "3~4" is assigned 2 points, and "5~6" is assigned 3 points.

**Total: PSQI total score=A+ B+C+D+ E+F+G**

Scoring reference:

| **PSQI total score** | **Sleep Quality** |
| --- | --- |
| 0-5 | Good sleep quality |
| 6-10 | Mild sleep quality |
| 11-15 | Poor sleep quality |
| 16-21 | Serious sleep quality |

**References**

**1.** Worldwide trends in hypertension prevalence and progress in treatment and control from 1990 to 2019: a pooled analysis of 1201 population-representative studies with 104 million participants. *Lancet.* 2021;398(10304):957-980.

**2.** Ungvari Z, Toth P, Tarantini S, et al. Hypertension-induced cognitive impairment: from pathophysiology to public health. *Nat Rev Nephrol.* 2021;17(10):639-654.

**3.** Escobar E. Hypertension and coronary heart disease. *J Hum Hypertens.* 2002;16 Suppl 1:S61-S63.

**4.** Ruiz-Ortega M, Rayego-Mateos S, Lamas S, Ortiz A, Rodrigues-Diez RR. Targeting the progression of chronic kidney disease. *Nat Rev Nephrol.* 2020;16(5):269-288.

**5.** Padwal R, Wood PW. Digital Health Approaches for the Assessment and Optimisation of Hypertension Care Provision. *Can J Cardiol.* 2021;37(5):711-721.

**6.** Dzau VJ, Balatbat CA. Future of Hypertension. *Hypertension.* 2019;74(3):450-457.

**7.** McManus RJ, Mant J, Franssen M, et al. Efficacy of self-monitored blood pressure, with or without telemonitoring, for titration of antihypertensive medication (TASMINH4): an unmasked randomised controlled trial. *Lancet.* 2018;391(10124):949-959.

**8.** McManus RJ, Little P, Stuart B, et al. Home and Online Management and Evaluation of Blood Pressure (HOME BP) using a digital intervention in poorly controlled hypertension: randomised controlled trial. *BMJ.* 2021;372:m4858.

**9.** Health WHOM, OL TMTE, Health-through-mobile-technologies. HWMC.

**10.** Kario K, Harada N, Okura A. Digital Therapeutics in Hypertension: Evidence and Perspectives. *Hypertension.* 2022;79(10):2148-2158.

**11.** Margolis KL, Asche SE, Bergdall AR, et al. Effect of home blood pressure telemonitoring and pharmacist management on blood pressure control: a cluster randomized clinical trial. *JAMA.* 2013;310(1):46-56.

**12.** Chen X, Zhou X, Li H, Li J, Jiang H. The value of WeChat application in chronic diseases management in China. *Comput Methods Programs Biomed.* 2020;196:105710.

**13.** Pletcher MJ, Fontil V, Modrow MF, et al. Effectiveness of Standard vs Enhanced Self-measurement of Blood Pressure Paired With a Connected Smartphone Application: A Randomized Clinical Trial. *JAMA Intern Med.* 2022;182(10):1025-1034.

**14.** McCartney DE, McManus RJ. Self-monitoring and self-management: new interventions to improve blood pressure control. *Curr Opin Nephrol Hypertens.* 2016;25(6):502-507.

**15.** McKinstry B, Hanley J, Wild S, et al. Telemonitoring based service redesign for the management of uncontrolled hypertension: multicentre randomised controlled trial. *BMJ.* 2013;346:f3030.

**16.** Uhlig K, Patel K, Ip S, Kitsios GD, Balk EM. Self-measured blood pressure monitoring in the management of hypertension: a systematic review and meta-analysis. *Ann Intern Med.* 2013;159(3):185-194.

**17.** Puddey IB, Beilin LJ, Vandongen R. Regular alcohol use raises blood pressure in treated hypertensive subjects. A randomised controlled trial. *Lancet.* 1987;1(8534):647-651.

**18.** Fuchs FD, Fuchs SC. The Effect of Alcohol on Blood Pressure and Hypertension. *Curr Hypertens Rep.* 2021;23(10):42.

**19.** Zhao F, Liu Q, Li Y, Feng X, Chang H, Lyu J. Association between alcohol consumption and hypertension in Chinese adults: Findings from the CHNS. *Alcohol.* 2020;83:83-88.

**20.** Valenzuela PL, Carrera-Bastos P, Galvez BG, et al. Lifestyle interventions for the prevention and treatment of hypertension. *Nat Rev Cardiol.* 2021;18(4):251-275.

**21.** Lewey J, Murphy S, Zhang D, et al. Effectiveness of a Text-Based Gamification Intervention to Improve Physical Activity Among Postpartum Individuals With Hypertensive Disorders of Pregnancy: A Randomized Clinical Trial. *JAMA Cardiol.* 2022;7(6):591-599.

**22.** Kario K, Nomura A, Harada N, et al. Efficacy of a digital therapeutics system in the management of essential hypertension: the HERB-DH1 pivotal trial. *Eur Heart J.* 2021;42(40):4111-4122.

**23.** Wang Y, Feng L, Zeng G, et al. Effects of Cuisine-Based Chinese Heart-Healthy Diet in Lowering Blood Pressure Among Adults in China: Multicenter, Single-Blind, Randomized, Parallel Controlled Feeding Trial. *Circulation.* 2022;146(4):303-315.

**24.** Semlitsch T, Jeitler K, Berghold A, et al. Long-term effects of weight-reducing diets in people with hypertension. *Cochrane Database Syst Rev.* 2016;3(3):D8274.

**25.** Baer HJ, Rozenblum R, De La Cruz BA, et al. Effect of an Online Weight Management Program Integrated With Population Health Management on Weight Change: A Randomized Clinical Trial. *JAMA.* 2020;324(17):1737-1746.

**26.** Hermida RC, Crespo JJ, Dominguez-Sardina M, et al. Bedtime hypertension treatment improves cardiovascular risk reduction: the Hygia Chronotherapy Trial. *Eur Heart J.* 2020;41(48):4565-4576.

**27.** Li C, Shang S. Relationship between Sleep and Hypertension: Findings from the NHANES (2007-2014). *Int J Environ Res Public Health.* 2021;18(15).

**28.** Araya R, Menezes PR, Claro HG, et al. Effect of a Digital Intervention on Depressive Symptoms in Patients With Comorbid Hypertension or Diabetes in Brazil and Peru: Two Randomized Clinical Trials. *JAMA.* 2021;325(18):1852-1862.

**29.** Morawski K, Ghazinouri R, Krumme A, et al. Association of a Smartphone Application With Medication Adherence and Blood Pressure Control: The MedISAFE-BP Randomized Clinical Trial. *JAMA Intern Med.* 2018;178(6):802-809.

**30.** Magid DJ, Olson KL, Billups SJ, Wagner NM, Lyons EE, Kroner BA. A pharmacist-led, American Heart Association Heart360 Web-enabled home blood pressure monitoring program. *Circ Cardiovasc Qual Outcomes.* 2013;6(2):157-163.

**31.** Bobrow K, Farmer AJ, Springer D, et al. Mobile Phone Text Messages to Support Treatment Adherence in Adults With High Blood Pressure (SMS-Text Adherence Support [StAR]): A Single-Blind, Randomized Trial. *Circulation.* 2016;133(6):592-600.

**32.** Bennell KL, Lawford BJ, Keating C, et al. Comparing Video-Based, Telehealth-Delivered Exercise and Weight Loss Programs With Online Education on Outcomes of Knee Osteoarthritis : A Randomized Trial. *Ann Intern Med.* 2022;175(2):198-209.

**33.** He FJ, Zhang P, Luo R, et al. App based education programme to reduce salt intake (AppSalt) in schoolchildren and their families in China: parallel, cluster randomised controlled trial. *BMJ.* 2022;376:e66982.

**34.** Neal B, Wu Y, Feng X, et al. Effect of Salt Substitution on Cardiovascular Events and Death. *N Engl J Med.* 2021;385(12):1067-1077.

**35.** Wilkinson MJ, Manoogian E, Zadourian A, et al. Ten-Hour Time-Restricted Eating Reduces Weight, Blood Pressure, and Atherogenic Lipids in Patients with Metabolic Syndrome. *Cell Metab.* 2020;31(1):92-104.

**36.** Lavie CJ, Milani RV, Ventura HO. Obesity and cardiovascular disease: risk factor, paradox, and impact of weight loss. *J Am Coll Cardiol.* 2009;53(21):1925-1932.

**37.** Zhao Y, Liu Y, Sun H, et al. Association of long-term dynamic change in body weight and incident hypertension: The Rural Chinese Cohort Study. *Nutrition.* 2018;54:76-82.

**38.** Kario K, Wang JG. Could 130/80 mm Hg Be Adopted as the Diagnostic Threshold and Management Goal of Hypertension in Consideration of the Characteristics of Asian Populations? *Hypertension.* 2018;71(6):979-984.

**39.** Zung WW. A rating instrument for anxiety disorders. *Psychosomatics.* 1971;12(6):371-379.

**40.** ZUNG WW. A SELF-RATING DEPRESSION SCALE. *Arch Gen Psychiatry.* 1965;12:63-70.

**41.** Buysse DJ, Reynolds CR, Monk TH, Berman SR, Kupfer DJ. The Pittsburgh Sleep Quality Index: a new instrument for psychiatric practice and research. *Psychiatry Res.* 1989;28(2):193-213.

**42.** Samadian F, Dalili N, Jamalian A. Lifestyle Modifications to Prevent and Control Hypertension. *Iran J Kidney Dis.* 2016;10(5):237-263.
