# Supplementary material for: Efficacy of a WeChat-Based Multimodal Digital Transformation Management Model in New-Onset Mild to Moderate Hypertension: Randomized Clinical Trial
Source: J Med Internet Res. 2023 Dec 4;25:e52464. doi: 10.2196/52464 (PMC10728790; doi:10.2196/52464)

**Table S1.** Home blood pressure reduction from baseline.

|  | **Intervention**  **(n = 88)** | | **Usual Care**  **(n = 87)** | |  |  |
| --- | --- | --- | --- | --- | --- | --- |
|  | **Mean**  **(95% CI)** | **BP change**  **(95% CI)** | **Mean**  **(95% CI)** | **BP change**  **(95% CI)** | **Difference change from baseline, mean**  **(95% CI)** | **P Value** |
| **SBP** |  |  |  |  |  |  |
| Baseline | 151.74  (150.04–153.44) |  | 150.78  (149.11–152.46) |  |  | 0.427 |
| 3 months | 128.81  (127.17–130.44) | -22.93  (-24.86 to -21.01) | 134.52  (132.39–136.65) | -16.26  (-18.72 to -13.81) | -6.67  (-9.76 to -3.57) | <0.001 |
| 6 months | 126.19  (124.40–127.98) | -25.55  (-27.95 to -23.14) | 133.48  (131.17–135.80) | -17.30  (-19.83 to -14.77) | -8.25  (-11.71 to -4.78) | <0.001 |
| **DBP** |  |  |  |  |  |  |
| Baseline | 94.22  (92.24–96.19) |  | 91.53  (89.45–93.61) |  |  | 0.064 |
| 3 months | 86.16  (84.24–88.08) | -8.06  (-10.23 to -5.88) | 87.35  (85.26–89.43) | -4.18  (-6.92 to -1.44) | -3.87  (-7.34 to -0.40) | 0.029 |
| 6 months | 82.28  (80.32–84.23) | -11.93  (-14.35 to -9.51) | 84.45  (82.49–86.41) | -7.08  (-9.72 to -4.44) | -4.85  (-8.41 to -1.30) | 0.008 |

Abbreviations: CI, confidence interval; SBP, systolic blood pressure; DBP, diastolic blood pressure

**Table S2.** Office blood pressure reduction from baseline.

|  | **Intervention**  **(n = 88)** | | **Usual Care**  **(n = 87)** | |  |  |
| --- | --- | --- | --- | --- | --- | --- |
|  | **Mean**  **(95% CI)** | **BP change**  **(95% CI)** | **Mean**  **(95% CI)** | **BP change**  **(95% CI)** | **Difference change from baseline, mean**  **(95% CI)** | **P Value** |
| **SBP** |  |  |  |  |  |  |
| Baseline | 153.64  (151.86–155.41) |  | 151.48  (149.96–153.00) |  |  | 0.069 |
| 3 months | 129.26  (127.47–131.05) | -24.38  (-26.46 to -22.29) | 136.67  (134.43–138.90) | -14.82  (-17.34 to -12.30) | -9.56  (-12.80 to -6.31) | <0.001 |
| 6 months | 127.81  (126.10–129.51) | -25.83  (-28.22 to -23.44) | 134.92  (132.76–137.06) | -16.56  (-18.95 to -14.18) | -9.27  (-12.62 to -5.91) | <0.001 |
| **DBP** |  |  |  |  |  |  |
| Baseline | 93.56  (91.77–95.35) |  | 91.31  (89.26–93.36) |  |  | 0.102 |
| 3 months | 85.60  (83.81–87.40) | -7.95  (-10.02 to -5.89) | 86.41  (84.54–88.29) | -4.90  (-7.54 to -2.25) | -3.06  (-6.38–0.27) | 0.072 |
| 6 months | 82.16  (80.45–83.87) | -11.40  (-13.64 to -9.16) | 85.09  (83.33–86.85) | -6.22  (-8.66 to -3.78) | -5.18  (-8.47 to -1.89) | 0.002 |

Abbreviations: CI, confidence interval; SBP, systolic blood pressure; DBP, diastolic blood pressure

**Table S3.** Composite and home blood pressure control.

|  | **Group** | **Intervention**  **(n = 88)** | **Usual Care**  **(n = 87)** | **χ^2^** | **P value** | **OR**  **(95% CI)** |
| --- | --- | --- | --- | --- | --- | --- |
| **Control rates of BP**  **(BP <140/90 mmHg)** | |  |  |  |  |  |
| 1 month | No | 34 (38.6) | 38 (43.7) | 0.459 | 0.498 | 1.232 (0.674–2.251) |
|  | Yes | 54 (61.4) | 49 (56.3) |  |  |  |
| 2 months | No | 46 (52.3) | 53 (60.9) | 1.331 | 0.249 | 1.423 (0.781–2.594) |
|  | Yes | 42 (47.7) | 34 (39.1) |  |  |  |
| 3 months | No | 43 (48.9) | 45 (51.7) | 0.143 | 0.705 | 1.121 (0.620–2.029) |
|  | Yes | 45 (51.1) | 42 (48.3) |  |  |  |
| 4 months | No | 40 (45.5) | 43 (49.4) | 0.277 | 0.599 | 1.173 (0.648–2.124) |
|  | Yes | 48 (54.5) | 44 (50.6) |  |  |  |
| 5 months | No | 28 (31.8) | 40 (46.0) | 3.691 | 0.055 | 1.483 (0.756–2.910) |
|  | Yes | 60 (68.2) | 47 (54.0) |  |  |  |
| 6 months | No | 24 (27.3) | 41 (47.1) | 7.386 | 0.007 | 2.377 (1.265–4.464) |
|  | Yes | 64 (72.7) | 46 (52.9) |  |  |  |
| **Control rates of BP**  **(BP <130/80 mmHg)** | |  |  |  |  |  |
| 1 month | No | 76 (86.4) | 80 (92.0) | 1.413 | 0.235 | 1.805 (0.675–4.825) |
|  | Yes | 12 (13.6) | 7 (8.0) |  |  |  |
| 2 months | No | 77 (87.5) | 79 (90.8) | 0.494 | 0.482 | 1.411 (0.538–3.696) |
|  | Yes | 11 (12.5) | 8 (9.2) |  |  |  |
| 3 months | No | 75 (85.2) | 77 (88.5) | 0.412 | 0.521 | 1.335 (0.552–3.229) |
|  | Yes | 13 (14.8) | 10 (11.5) |  |  |  |
| 4 months | No | 73 (83.0) | 74 (85.1) | 0.144 | 0.704 | 1.170 (0.520–2.629) |
|  | Yes | 15 (17.0) | 13 (14.9) |  |  |  |
| 5 months | No | 61 (69.3) | 67 (77.0) | 1.318 | 0.251 | 1.483 (0.756–2.910) |
|  | Yes | 27 (30.7) | 20 (23.0) |  |  |  |
| 6 months | No | 56 (63.6) | 71 (81.6) | 7.100 | 0.008 | 2.536 (1.266–5.080) |
|  | Yes | 32 (36.4) | 16 (18.4) |  |  |  |

Values are given as n (%).

Abbreviations: OR, odds ratio; CI, confidence interval; BP, blood pressure

**Table S4.** Subgroup analysis of 6-month blood pressure control rates (<140/90 mmHg).

|  | **Intervention**  **(n = 88)** | **Usual Care**  **(n = 87)** | **OR (95% CI)** | **Interaction P value** |
| --- | --- | --- | --- | --- |
| **Sex** |  |  |  | 0.088 |
| Male | 47 | 47 | 5.228 (2.070–13.204) |  |
| Female | 41 | 40 | 1.040 (0.422–2.563) |  |
| **Age** |  |  |  |  |
| <60 years | 60 | 60 | 2.070 (0.970–4.415) | 0.012 |
| ≥60 years | 28 | 27 | 3.231 (1.032–10.111) |  |
| **Educational qualifications** |  |  |  |  |
| Elementary school and below | 22 | 18 | 1.364 (0.370–5.028) | 0.013 |
| Middle school or high school | 34 | 28 | 3.750 (1.266–11.110) |  |
| Bachelor degree and above | 32 | 41 | 2.207 (0.824–5.911) |  |
| **BMI** |  |  |  |  |
| <30 (kg/m^2^) | 78 | 80 | 2.190 (1.131–4.240) | 0.008 |
| ≥30 (kg/m^2^) | 10 | 7 | 5.333 (0.618–45.991) |  |
| **Diabetes mellitus** |  |  |  |  |
| No | 66 | 72 | 1.946 (0.966–3.920) | 0.058 |
| Yes | 2 | 15 | 5.143 (1.166–22.687) |  |
| **Coronary artery disease** |  |  |  |  |
| No | 81 | 74 | 2.455 (1.241–4.855) | 0.276 |
| Yes | 7 | 13 | 1.200 (0.185–7.770) |  |
| **Current smoking** |  |  |  |  |
| No | 62 | 65 | 1.573 (0.767–3.225) | 0.008 |
| Yes | 26 | 22 | 9.200 (2.122–39.889) |  |
| **Current drinking** |  |  |  |  |
| No | 60 | 60 | 1.541 (0.730–3.256) | 0.027 |
| Yes | 28 | 27 | 6.691 (1.946–23.000) |  |
| **Baseline SBP** |  |  |  |  |
| <160 mmHg | 70 | 72 | 3.020 (1.463–6.234) | 0.500 |
| ≥160 mmHg | 18 | 15 | 1.094 (0.276–4.330) |  |

Abbreviations: OR, odds ratio; CI, confidence interval; BMI, body mass index; SBP, systolic blood pressure

**Table S5.** Subgroup analysis of 6-month blood pressure control rates (<130/80 mmHg).

|  | **Intervention**  **(n = 88)** | **Usual Care**  **(n = 87)** | **OR (95% CI)** | **Interaction P value** |
| --- | --- | --- | --- | --- |
| **Sex** |  |  |  | 0.042 |
| Male | 47 | 47 | 2.621 (1.029–6.674) |  |
| Female | 41 | 40 | 2.444 (0.864–6.917) |  |
| **Age** |  |  |  | 0.077 |
| <60 years | 60 | 60 | 1.549 (0.678–3.539) |  |
| ≥60 years | 28 | 27 | 8.000 (1.952–32.782) |  |
| **Educational qualifications** |  |  |  | 0.277 |
| Elementary school and below | 22 | 18 | 4.571 (0.829–25.211) |  |
| Middle school or high school | 34 | 28 | 3.273 (0.918–11.664) |  |
| Bachelor degree and above | 32 | 41 | 1.860 (0.677–5.107) |  |
| **BMI** |  |  |  | 0.070 |
| <30 (kg/m^2^) | 78 | 80 | 2.294 (1.106–4.760) |  |
| ≥30 (kg/m^2^) | 10 | 7 | 6.000 (0.516-69.754) |  |
| **Diabetes mellitus** |  |  |  | 0.307 |
| No | 66 | 72 | 1.973 (0.889–4.381) |  |
| Yes | 2 | 15 | 4.800 (1.052–21.907) |  |
| **Coronary artery disease** |  |  |  | 0.086 |
| No | 81 | 74 | 2.439 (1.184–5.022) |  |
| Yes | 7 | 13 | 2.000 (0.106–37.830) |  |
| **Current smoking** |  |  |  |  |
| No | 62 | 65 | 1.536 (0.659–3.581) | 0.249 |
| Yes | 26 | 22 | 7.200 (1.884–27.522) |  |
| **Current drinking** |  |  |  | 0.732 |
| No | 60 | 60 | 2.500 (1.052–5.940) |  |
| Yes | 28 | 27 | 2.625 (0.810–8.510) |  |
| **Baseline SBP** |  |  |  | 0.252 |
| <160 mmHg | 70 | 72 | 2.601 (1.221–5.543) |  |
| ≥160 mmHg | 18 | 15 | 2.500 (0.409–15.293) |  |

Abbreviations: OR, odds ratio; CI, confidence interval; SBP, systolic blood pressure

**Table S6.** Quality-of-life total score reduction 6 months from baseline.

|  | **Intervention**  **(n = 88)** | | **Usual Care**  **(n = 87)** | |  |  |
| --- | --- | --- | --- | --- | --- | --- |
|  | **Mean**  **(95% CI)** | **Score change**  **(95% CI)** | **Mean**  **(95% CI)** | **Score change**  **(95% CI)** | **Difference change from baseline, mean**  **(95% CI)** | P Value |
| SDS scores |  |  |  |  |  |  |
| Baseline | 47.42 (45.73–49.11） |  | 45.69 (44.03–47.35) |  |  | 0.148 |
| 6 months | 40.92 (39.25–42.59） | -6.50 (-7.52 to -5.48) | 43.56 (41.79–45.33) | -2.13 (-2.98 to -1.28) | -4.41 (-5.76 to -3.07) | 0.032 |
| SAS scores |  |  |  |  |  |  |
| Baseline | 51.51 (49.49–53.53） |  | 49.35 (46.99–51.70) |  |  | 0.167 |
| 6 months | 42.66 (40.77–44.55） | -8.85 (-10.01 to -7.70) | 45.76 (43.54–47.97) | -3.59 (-4.68 to -2.49) | -5.34 (-6.98 to -3.71) | 0.036 |
| PSQI scores |  |  |  |  |  |  |
| Baseline | 8.76 (8.20–9.32） |  | 9.32 (8.60–10.05) |  |  | 0.225 |
| 6 months | 7.32 (6.87–7.77） | -1.44 (-1.80 to -1.08) | 8.89 (8.28–9.49) | -0.44 (-0.84 to -0.04) | -1.00 (-1.53 to -0.47) | <0.001 |

Abbreviations: CI, confidence interval; SAS, self-rating anxiety scale; SDS, self-rating depression scale; PSQI, the Pittsburgh Sleep Quality Index

**Table S7.** Number of people with improved adverse factors.

| **Lifestyle Factors (n, %)** | **Intervention**  **(n = 88)** | **Usual Care**  **(n = 87)** |
| --- | --- | --- |
| **Overweight/obesity improvement** | 8 (9.09%) | 2 (2.30%) |
| **Physical activity improvement (>2 h/week)** | 18 (20.45%) | 7 (8.05%) |
| **Quit smoking** | 4 (15.38%) | 1 (4.54%) |
| **Quit drinking** | 3 (10.71%) | NA |

Values are given as n (%).

**Table S8.** Newly initiated antihypertensive medication.

|  | **Intervention**  **（n** **=** **88）** | **Usual Care**  **（n** **=** **87）** |
| --- | --- | --- |
| **Antihypertensive medication*, n (%)** |  |  |
| **1** | 37 | 34 |
| **2** | 3 | 6 |
| **>2** | 4 | 4 |
| **Compound preparation** | 7 | 9 |
| **Drug type for monotherapy, n (%)** |  |  |
| **CCB** | 23 | 26 |
| **ACEI** | 7 | 6 |
| **ARB** | 5 | 3 |
| **β-blockers** | 2 | 1 |
| **Diuretics** | NA | NA |

Abbreviations: CCB, calcium channel blocker; ACEI, angiotensin-converting enzyme inhibitor; ARB, angiotensin receptor blocker

**Table S9.** Adverse events in the study.

| **Adverse Event [No. (%)]** | **Intervention**  **（n=88）** | **Usual Care**  **（n=87）** |
| --- | --- | --- |
| CVD Event |  |  |
| AMI | NA | NA |
| PCI | NA | NA |
| Stroke | NA | NA |
| Death | NA | NA |
| Adverse Event |  |  |
| Cough | NA | NA |
| Stomach discomfort | 1 (1.14%) | 1 (1.15%) |
| Edema | NA | NA |
| Dizziness | 2 (2.27%) | 3 (3.45%) |
| Other | NA | NA |

Abbreviations: AMI, acute myocardial infraction; PCI, Percutaneous Coronary Intervention; CVD, cardiovascular disease; NA, no data available.


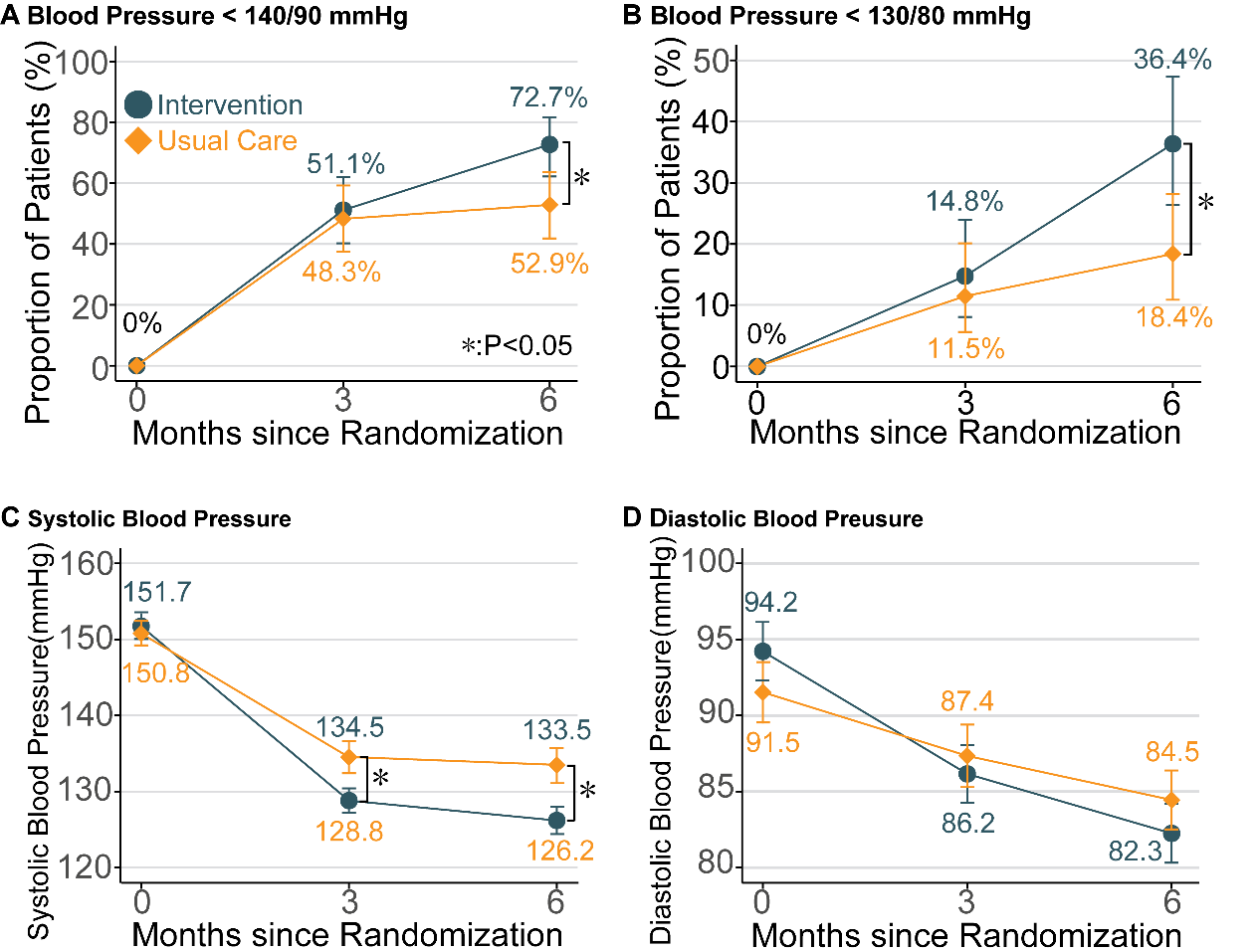
**Figure S1**. Home blood pressure control during trial follow-up in intervention and control groups.

Proportion of patients with systolic blood pressure <140 mm Hg and diastolic blood pressure <90 mm Hg (A) and those with systolic blood pressure <130 mm Hg and diastolic blood pressure <80 mm Hg (B). Mean systolic blood pressure (C) and mean diastolic blood pressure (D). I bars indicate 95% confidence intervals.* indicate p value <0.05.

**Figure S2**. Office blood pressure control during trial follow-up in intervention and control groups.


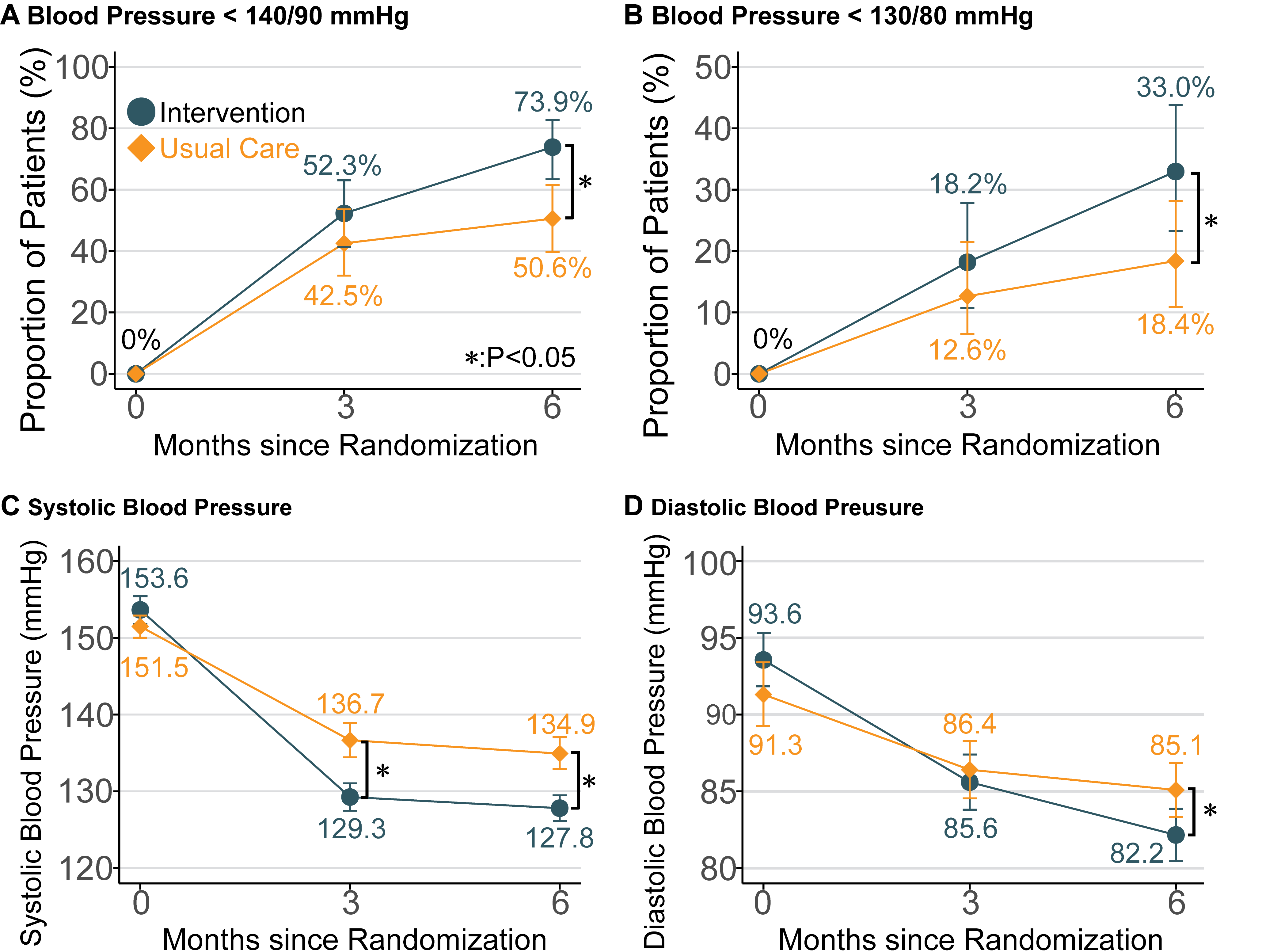


Proportion of patients with systolic blood pressure <140 mm Hg and diastolic blood pressure <90 mm Hg (A) and those with systolic blood pressure <130 mm Hg and diastolic blood pressure <80 mm Hg (B). Mean systolic blood pressure (C) and mean diastolic blood pressure (D). I bars indicate 95% confidence intervals.* indicate p value <0.05.

**Figure S3**. Visual summary of multimodal management of hypertension.


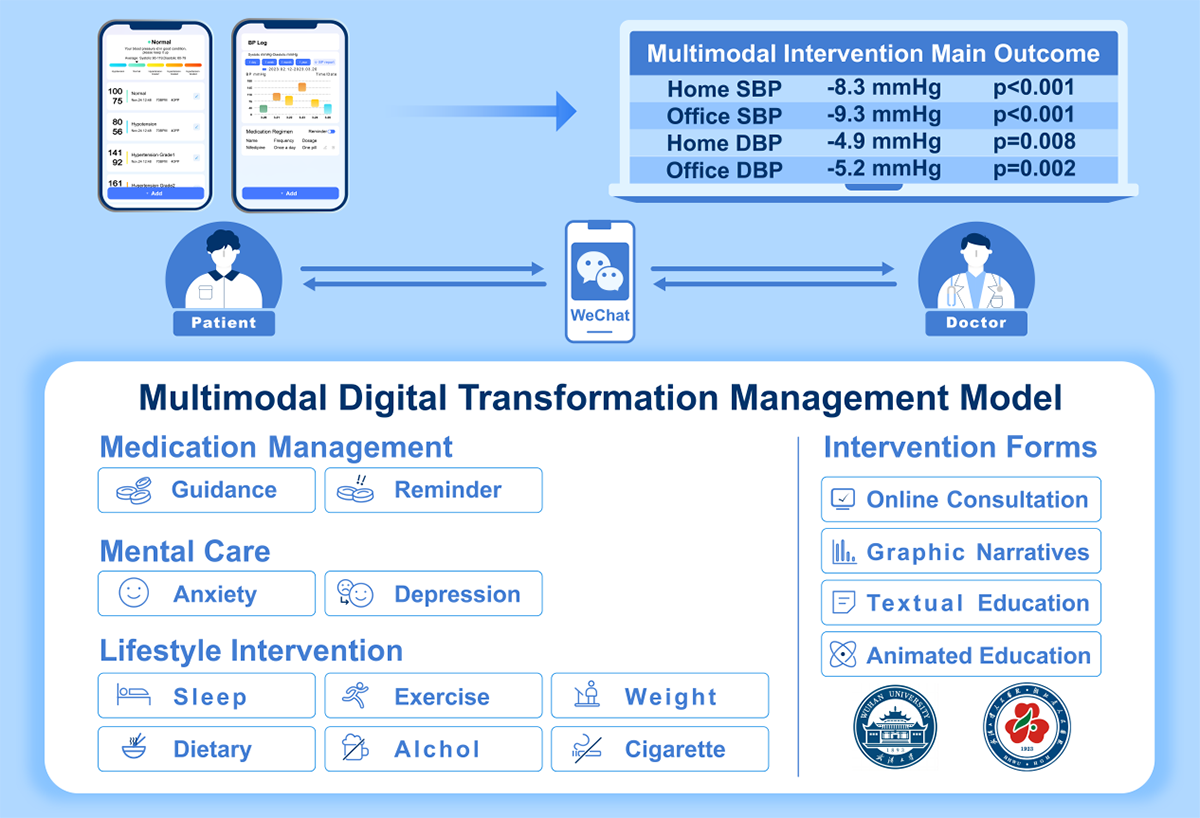

Supplement: Multimedia Appendix 2 [file jmir_v25i1e52464_app2.docx]
